# Flexibility in data interpretation: effects of representational format

**DOI:** 10.3389/fpsyg.2013.00980

**Published:** 2013-12-31

**Authors:** David W. Braithwaite, Robert L. Goldstone

**Affiliations:** Percepts-Concepts Laboratory, Department of Psychological and Brain Sciences, Indiana UniversityBloomington, IN, USA

**Keywords:** representations, flexibility, graphs, tables, statistics instruction

## Abstract

Graphs and tables differentially support performance on specific tasks. For tasks requiring reading off single data points, tables are as good as or better than graphs, while for tasks involving relationships among data points, graphs often yield better performance. However, the degree to which graphs and tables support flexibility across a range of tasks is not well-understood. In two experiments, participants detected main and interaction effects in line graphs and tables of bivariate data. Graphs led to more efficient performance, but also lower flexibility, as indicated by a larger discrepancy in performance across tasks. In particular, detection of main effects of variables represented in the graph legend was facilitated relative to detection of main effects of variables represented in the x-axis. Graphs may be a preferable representational format when the desired task or analytical perspective is known in advance, but may also induce greater interpretive bias than tables, necessitating greater care in their use and design.

## Introduction

Humans employ external representations of information, such as graphs, diagrams, tables, and equations, to assist in performing a wide variety of tasks in a wide variety of domains. Often, the same information may be represented using more than one representational format. Two different representations of the same information are said to be *informationally equivalent* if either can be reconstructed perfectly on the basis of the other (Simon, 1978). Even when two representations are informationally equivalent, some tasks may be performed more accurately or efficiently with one than the other (Larkin and Simon, 1987). Thus, extensive research has been devoted to understanding the relative strengths and weaknesses of different representational formats for different tasks.

Many representational formats may be classified as either *graphical* or *symbolic*. Graphical representations, such as graphs and diagrams, use spatial features such as height and distance to convey information, while symbolic representations, such as tables and equations, employ formal symbols such as Arabic numerals and algebraic symbols for the same purpose. The distinction is relative rather than absolute: Symbols such as numerals may appear in graphs, and spatial information such as row or column location may convey information in tables. For an in-depth discussion of types of representational format, see Stenning (2002; see also Paivio, 2007).

The present study aims to contribute to a deeper understanding of the relative advantages of one common graphical format, Cartesian statistical graphs (henceforth simply “graphs”), and one common symbolic format, tables. Understanding the relative utility of graphs and tables is important from a practical viewpoint because of the extensive use of both representational formats for data analysis and communication in fields such as science, business, and education. The comparison between graphs and tables is also interesting from a theoretical point of view because they are often informationally equivalent in the sense described above. As such, differences between these formats in accuracy or efficiency of task performance may be attributed to the effects of representational format *per se*, rather than to differences in informational content. Thus, understanding how task performance differs depending on whether graphs or tables are used can support a more general theory of the cognitive effects of external representational format.

Extensive research has yielded fairly clear conclusions regarding the relative strengths of graphs and tables for a variety of individual tasks. In general, tasks that can be performed by reading individual data points are performed with tables as well as or better than with graphs (Vessey and Galletta, 1991; Coll, 1992; Meyer et al., 1999; Meyer, 2000; Porat et al., 2009). In an early study of Vessey and Galletta (1991), accuracy was higher and reaction time faster with tables than with line graphs for tasks requiring participants to read off individual values of a dependent variable for specified levels of two independent variables. A more recent study of Porat et al. (2009) found no difference in performance between graphs and tables for a task which could be achieved by attending to individual data points, namely detecting increases in amplitude of periodic functions (such increases may be detected by attending to any of the function's maxima).

By contrast, many studies have shown clear advantages for graphs over tables for tasks involving relationships among multiple data points (Vessey and Galletta, 1991; Meyer et al., 1999; Porat et al., 2009; Schonlau and Peters, 2012; but see Meyer, 2000, for a contrary result). Performance was better with graphs than with tables for tasks requiring participants to compare differences between pairs of data points in the study of Vessey and Galletta (1991) mentioned above, and for tasks requiring comparing differences between multiple adjacent points in that of Porat et al. (2009). Consistent with the relative importance of relationships between points, rather than values of individual points, in graph reading, Maichle (1994) found that skilled graph readers tended to focus spontaneously on data trends rather than individual data points when interpreting graphs, and required more effort to recover information about the latter than about the former.

Models of graph comprehension (Pinker, 1990; Carpenter and Shah, 1998; Freedman and Shah, 2002; Trickett and Trafton, 2006; Ratwani et al., 2008) suggest a possible explanation for the relative advantage of graphs on tasks involving relationships among data points. While these models differ in many respects, they generally agree that the process of graph comprehension includes at least the following processes: encoding of visual features such as spatial patterns, interpreting these features using prior knowledge, and relating the interpretations back to the graph referents, such as the specific variables represented by the graph axes. Visual features are, therefore, the raw material on which graph interpretation processes operate (Pinker, 1990). “Features” is employed here in a broad sense (Tversky, 1977), including simple visual relations such as distances between points as well as complex visual patterns such as curving or crossing lines.

Relations between multiple data points can be represented in graphs by single visual features. For example, the difference in values represented by two bars in a bar graph may be ascertained from the vertical distance between the tops of the bars, the rate of acceleration in a function graph from the degree of curvature of the graph line, and so on. Importantly, it may be possible for graph readers to encode and, subsequently, interpret such visual features as single units, without separately encoding and interpreting the individual data points they comprise (Pinker, 1990; Pomerantz and Portillo, 2012). Even complex visual features may be detected efficiently through automatized visual routines (Ullman, 1984). Consistent with this view, eye-tracking studies provide evidence that graph readers are sensitive to complex visual elements such as distinct functional forms in line graphs (Carpenter and Shah, 1998) or similarly-colored clusters in choropleth graphs (Ratwani et al., 2008). Thus, graphs may offer a shortcut to the recognition of relationships among data points. Such shortcuts are likely unavailable in the case of tables, in which visual patterns like those just mentioned are either absent or far less salient than in graphs. Thus, the relative advantage of graphs for recognition of relationships among data points may reflect a facilitatory function of visual patterns for the process of graph comprehension, which has no analog in the process of table comprehension. In short, in graphs, relationships can often be depicted by easily encoded visual features.

While much is already known about the relative advantages of graphs and tables for performing various *individual* tasks, less is known about their relative advantages with respect to flexibility across a *range* of different tasks. “Flexibility” refers, here, to the ability to perform different tasks equally well. Equally good performance across tasks would indicate high flexibility, while lower performance on some tasks than on others would indicate low flexibility. How might representational format affect flexibility?

To make this question concrete, we introduce the stimuli and tasks employed in the present study. The stimuli were representations of simulated results from fictional experiments. Each fictional experiment involved two binary independent variables, one representing an experimentally manipulated treatment, and the other representing an observed demographic variable assumed to be of secondary interest. For generality across different stimuli, the terms “treatment factor” and “secondary factor” will be used to refer to the first and second types of variable, respectively.

**Figure 1:**
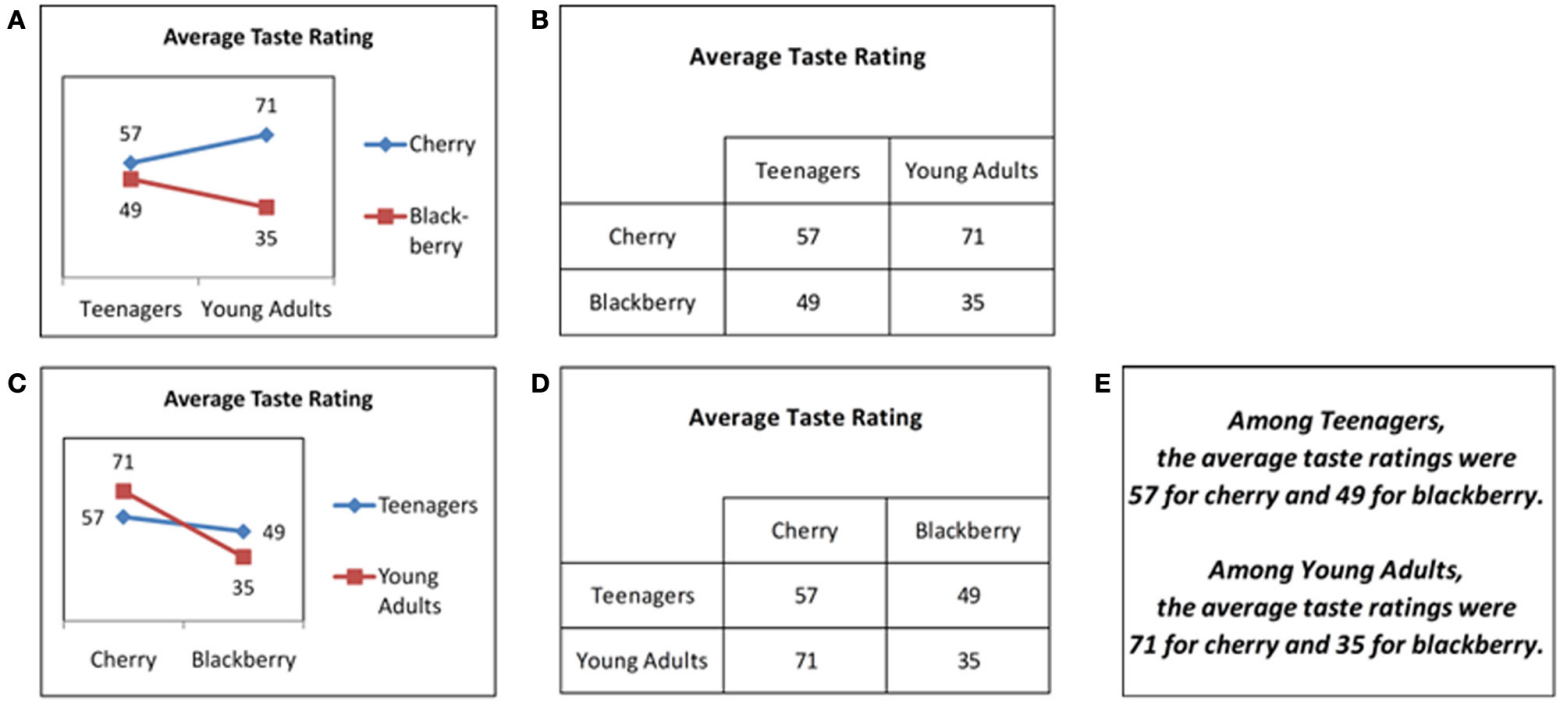
**Test stimuli showing possible outcomes of a fictional taste test study. (A)** Graphical format, Experiment 1. **(B)** Tabular format, Experiment 1. **(C)** Graphical format, Experiment 2. **(D)** Tabular format, Experiment 2. **(E)** Text format, Experiments 1–2.

Example stimuli are shown in Figures 1A–E. These figures show simulated results of a taste test study soliciting likeability ratings for soft drinks. In these examples, the treatment factor is drink flavor, and the secondary factor is age group. Figures 1A–E all represent the same data, in graphical (Figures 1A,C), tabular (Figures 1B,D), or text (Figure 1E) format. The assignment of visual dimensions to factors varies between representations. For example, line color represents the treatment factor in Figure 1A, but the secondary factor in Figure 1C, while x-axis position represents the secondary factor in Figure 1A, but the treatment factor in Figure 1C. Similarly, table rows and table columns represent different factors' levels in Figures 1B,D. However, the assignments of visual dimensions to factors were fixed for all stimuli within each of the experiments reported below.

The tasks involved judging the presence or absence, in each simulated result, of three statistical effects: main effects of the treatment factor, main effects of the secondary factor, and interaction effects of the two factors. The corresponding tasks will be referred to as the treatment task, the secondary task, and the interaction task. Note that the treatment and secondary tasks are similar, in that the formal criteria for judging the presence of a main effect of a variable do not depend on whether that variable was experimentally manipulated, as for the treatment task, or observed, as for the secondary task. However, these tasks do differ for a given assignment of visual dimensions to factors. For example, if graph legends/table rows are assigned to represent treatment factors, and x-axes/table columns to represent secondary factors (Figures 1A,B), then the treatment and secondary tasks require attending to different visual dimensions in the stimuli and, therefore, do constitute different tasks.

In the context of these tasks, the idea of flexibility can be operationalized in two ways. The first involves comparing performance between different tasks performed with the same representation. For example, one might compare performance between the treatment and secondary tasks with the graph in Figure 1A, or again with the table in Figure 1B. The second way involves comparing performance on a given task across assignments of visual dimensions to factors. For example, one might compare performance on the treatment task between the graph in Figure 1A, which represents the treatment factor in the legend, and that in Figure 1C, which represents the treatment factor by x-axis position. Similarly, one might compare performance on the treatment task between the tables in Figures 1B,D. In either case, the smaller the difference in performance for a given representational format, the higher the flexibility of that format. The present study focuses on the first way of assessing flexibility, i.e., comparing performance between different tasks for a given representation, but the second way, i.e., comparing performance for a given task between alternate assignments of visual dimensions to factors, was also investigated in Experiment 2.

All three tasks involve relationships among data points. Therefore, an overall facilitatory effect might be expected from graphs relative to tables, for the reasons given in the previous sub-section. For example, separated graph lines (as in Figure 1A) suggest an effect of the legend variable, while consistent upward or downward slope for both graph lines (as in Figure 1C) suggests an effect of the x-axis variable. Once such associations between spatial configurations and statistical effects are learned, faster and more accurate recognition of such effects could be expected with graphs. Analogous cues are unavailable with tables, which might therefore lead to slower and/or less accurate performance. However, existing research is less clear on the question of whether graphs or tables would lead to greater flexibility that is, to smaller differences in performance across tasks. Two possibilities for how representational format might affect flexibility are discussed below.

The first possibility, termed “intrinsic bias,” is that a format might make certain tasks intrinsically easier at the expense of others, thus reducing flexibility. To illustrate this possibility, consider the treatment and secondary tasks. These tasks involve the same mathematical operations, i.e., calculating and comparing marginal means between levels of a designated factor, and so should be equally easy, absent effects of representational format. However, if representational properties relevant to one task are more salient than those relevant to another, the former task could be facilitated and the latter inhibited, resulting in bias favoring the former. For example, x-axis position might be more salient than line color in line graphs, or column position more salient than row position in two-dimensional tables. For graphs and tables like those in Figures 1A,B, such differences in salience would create bias favoring the secondary task relative to the treatment task, because the secondary factor is represented in the x-axis in Figure 1A and the table columns in Figure 1B. In both cases, such bias could be a cause of reduced flexibility across tasks.

Evidence suggests that such effects are likely to be present for graphs. Graph interpretation is influenced by attributes, such as color brightness that affect the visual salience of different representational dimensions (Hegarty et al., 2010). Moreover, the visual dimensions chosen to represent variables in graphs affects the ease with which particular tasks are performed (Gattis and Holyoak, 1996; Zacks et al., 1998; Peebles and Cheng, 2003; Hegarty et al., 2010; Kessell and Tversky, 2011; Tversky et al., 2012, 2013). More direct evidence comes from studies investigating interpretation of graphs of bivariate data (Shah and Carpenter, 1995; Carpenter and Shah, 1998; Shah and Freedman, 2011), which have consistently found that the choice of which variable to represent on the x-axis, and which in the graph legend, can strongly impact graph interpretation. For example, Shah and Freedman (2011) found that spontaneous interpretations of bivariate bar and line graphs with categorical independent variables tended to interpret interaction effects preferentially as mediating effects of the legend variable on effects of the x-axis variable, rather than vice versa. However, analogous effects on performance for predefined tasks, rather than spontaneous interpretations, have not been shown.

A more important limitation to existing evidence is the lack of research comparing graphs and tables with respect to the presence of bias favoring some tasks over others. However, there is some reason to think that such bias might be greater for graphs than for tables. In multivariate data, different variables are typically represented by quite different visual dimensions in graphs, but by similar visual dimensions in tables. For example, in the graph in Figure 1A, one factor is represented by a non-spatial dimension (line color) and the other by a spatial dimension (x-axis position), but in the table in Figure 1B, both factors are represented by spatial dimensions (row and column position). The greater difference in the visual dimensions used to represent different variables in graphs suggests that the ease of accessing information about each variable might also differ more for graphs than for tables. Furthermore, as discussed previously, complex visual patterns in graphs serve to convey information in a manner for which tables have no obvious analog. For example, various configurations, such as the sideways “v” shape in Figure 1A, indicate the presence of an effect of the legend variable. These visual patterns may vary in salience. If the patterns relevant to a particular task happen to be highly salient, performance of that task would be facilitated relative to other tasks. Thus, the importance of complex visual patterns for graph interpretation creates an additional opportunity for intrinsic bias toward specific tasks that is absent or reduced for tables.

The possibility of differences in salience of representational properties relevant to different tasks has the interesting implication that the more salient representational properties could influence performance even on tasks for which they are not relevant. For example, in line graphs, line slope is relevant for detecting effects of the x-axis variable, whereas differences in line heights are not relevant for this purpose, but are relevant for detecting effects of the legend variable. However, if differences in line heights are highly salient, their presence might affect judgments regarding effects of the x-axis variable, despite their irrelevance to such judgments. More generally, if representational properties relevant to judging the presence of a given effect are highly salient on average, the presence of that effect might be expected to influence performance on other tasks to which the given effect is irrelevant. The high salience of representational properties relevant to the given effect might also be expected to create bias in favor of the task associated with that effect. Thus, examining influence from each effect on performance of tasks to which it is irrelevant provides a means of understanding differences in salience of representational properties relevant to different tasks, which is a potential source of intrinsic bias. With this goal in mind, the concept of influence is formalized in the analyses presented subsequently.

The second possible way in which representational format might affect flexibility across tasks is termed “transfer of practice.” In general, “transfer” refers to any influence of experience with one task on subsequent performance of a different task (Lobato, 2006). Such influence could be positive if learners are able to improve performance on the later task by applying or adapting methods learned from experience with the earlier task. On the other hand, such influence could be negative if performance on the later task is inhibited by learners' persistence in applying, without adaptation, methods learned for the earlier task but inappropriate for the later task. These two types of effect are termed “positive transfer” and “negative transfer,” respectively.

In the present study, participants performed the treatment, interaction, and secondary tasks in separate blocks, always starting with the treatment task and ending with the secondary task, with a fixed assignment of visual dimensions to factors for each representational format. Facilitation of the secondary task by experience with the previous tasks, e.g., the treatment task, would constitute positive transfer, whereas inhibition of the secondary task by such experience would constitute negative transfer. In this context, positive transfer for a given representational format is presumed to result from flexible adaptation of previous learning, and therefore would indicate high flexibility. By contrast, negative transfer for a given format is presumed to indicate inappropriate persistence in applying previously learned methods, and therefore would indicate low flexibility. The specific effects that positive and negative transfer would have on task performance are complicated by details of the experimental methodology, so examination of such effects is deferred until the Discussion of Experiment 1.

Previous research offers no clear prediction regarding the likelihood of positive or negative transfer of practice between tasks for graphs. In general, practice with a given task is likely to increase learners' sensitivity to visual features of graphs that are relevant to performing that task. The effects of such increased sensitivity on subsequent performance of other tasks could be negative, neutral, or positive, depending on whether the learned features are relevant to the subsequent tasks. For example, assume the treatment factor to be represented by graph line color and the secondary factor by x-axis position, as in Figure 1A. In this case, the sideways “v” shape in Figure 1A would be a useful cue for the treatment task, because its presence signifies the presence of a treatment effect, but not for the secondary task, because its presence is consistent with either the presence or absence of a secondary effect^1^. Thus, if practice with the treatment task increased sensitivity to sideways “v” shapes, subsequent performance on the secondary task would likely not be facilitated, and might even be inhibited. On the other hand, line height is a useful visual feature for both the treatment and secondary tasks, because line height represents taste rating, which is relevant for both tasks. Thus, if practice with the treatment task increased sensitivity to line height, subsequent performance on the secondary task might be facilitated. In sum, whether graphs afford positive or negative transfer of practice between tasks may depend on the types of visual features relied upon during task performance.

To our knowledge, very few studies have explored the possibility of transfer of practice between tasks with graphs, and even fewer with tables. Suggestive evidence that transfer of practice may occur with graphs comes from studies finding positive effects of graph literacy on performance of specific graph interpretation tasks (Maichle, 1994; Shah and Freedman, 2011). For example, relative to less graph-literate participants, more graph-literate participants in Maichle's (1994) study demonstrated greater sensitivity to high-level visual features of graphs, i.e., configurations of data points, and paid more attention to labels. However, effects of graph literacy do not necessarily demonstrate transfer of practice between tasks, because graph literacy may in part reflect practice with the same graph interpretation tasks used in the studies. Very few studies have examined effects of experience with one task on performance of a different task in a controlled setting.

As an exception, Porat et al. (2009) had participants detect either increases or decreases in the amplitude of functions displayed in graphical or tabular format following practice performing either the same task (e.g., detecting increases following detecting increases) or a different task (e.g., detecting decreases following detecting increases) requiring a different strategy. When the second task was different from the first, but not when it was the same, participants showed poorer initial performance on the second task with tables than with graphs, suggesting higher flexibility with graphs. However, this effect was only found among a sub-group of participants selected based on strategy use displayed during the experiment, leaving open the possibility of selection bias. Thus, while suggestive, these findings require further validation. It is, additionally, desirable to investigate the question of flexibility over a wider range of representations and tasks.

To summarize, studies demonstrating differences in spontaneous attention to different visual dimensions in graphs (e.g., Shah and Freedman, 2011) and theories emphasizing the importance of complex visual configurations for graph reading (e.g., Pinker, 1990) both suggest the possibility of intrinsic bias in graphs, while there is presently no evidence that similar factors affect table reading. These considerations imply that graphs may be a less flexible representational format than tables, although they do not provide direct evidence for this view, due to the sparsity of relevant evidence for tables. On the other hand, there is limited evidence of greater positive transfer between tasks performed with graphs rather than with tables (Porat et al., 2009), implying that graphs may be the more flexible representational format. The present study was designed to investigate whether graphs or tables afford greater flexibility over a specific range of tasks and to evaluate intrinsic bias and transfer of practice as possible explanations for any observed differences in flexibility.

## Experiment 1

### Methods

#### Participants

Participants were *N* = 127 undergraduate students from the Indiana University Psychology Department who participated in partial fulfillment of a course requirement. Participants were approximately evenly split between males (*N* = 67) and females (*N* = 60). Most (*N* = 120) were aged 18–21, with the remainder (*N* = 7) aged 22 or older. The majority (*N* = 80) reported never having learned about main and interaction effects before the experiment. As reported previous experience had no effect on test accuracy, *p* = 0.947, it was not included as a factor in the subsequent analyses.

#### Design

The experiment employed a 3 × 3 × 3 mixed design with task (treatment, interaction, or secondary) and format (graph, table, or text) as within-subjects factors and training condition as a between-subjects factor. The different training conditions are not described in detail below, as the training manipulation had no significant effects or interactions on our dependent measures.

#### Materials

A set of tables (Figure 1A), graphs (Figure 1B), and text passages (Figure 1E) were developed as stimuli to be used for testing participants. The test stimuli showed possible results of a drink taste test involving two binary factors: drink flavor and age group. As mentioned in the Introduction, the term “treatment factor” is used to refer to the experimentally manipulated variable, i.e., drink flavor, and to the analogous factor in the training stimuli. Similarly, the term “secondary factor” is used to refer to the observed demographic variable assumed to be of secondary interest to the experimenter, i.e., age group, and to the analogous factor in the training stimuli. Main effects of the treatment and secondary factors are referred to as treatment and secondary effects, respectively, and interactions of both factors as interaction effects.

The test stimuli were developed as follows. First, 16 datasets were generated quasi-randomly, with two datasets representing each possible combination of presence or absence of each of the three effects just mentioned. The datasets thus generated included a wide variety of data configurations, e.g., main effects with and without interactions, interactions with and without interactions, both crossover and non-crossover interactions, etc. Importantly, each effect was present in exactly half of the datasets, and no effect's presence was correlated with the presence of any other effect. Next, three stimuli were created from each dataset by displaying the dataset in each of three formats: graph (Figure 1A), table (Figure 1B), and text (Figure 1E), yielding a total of 48 stimuli. While the training stimuli, described below, included both graphs and tables, text representations were only included in the test stimuli. Performance with the text format was intended primarily for assessing the relative effectiveness of different training conditions in promoting transfer. Importantly, the treatment factor was always displayed in the legends of the graphs and in the rows of the tables, leading to a vertical orientation in both cases, which was reversed in the case of the text passages. Similarly, the secondary factor was always displayed in the x-axis of the graphs and in the columns of the tables, leading to a horizontal orientation, which was again reversed in the text passages. The complete set of test stimuli is available in the Supplementary Materials.

A different fictional study, regarding effects of cognitive enhancers on test scores, was used as a basis for examples to be shown during training. This study, like that used to generate the test stimuli, involved two binary factors: drug type and participant sex, drug type being the treatment factor and participant sex the secondary factor. For each of the three effect types, two datasets were created, with the given effect present in one and absent in the other. One graph and one table were created for each dataset. Thus, there were four examples for each effect, including two examples illustrating the effect's presence and two illustrating its absence, with one graph and one table in each case. Critically, the same orientation conventions were used as for the test stimuli. Thus, for both the training and test stimuli, the treatment factor was displayed in the legend of the graphs and in the rows of the tables, while the secondary factor was displayed in the x-axis of the graphs and in the columns of the tables.

The decision to employ line graphs for the graph stimuli requires some justification. Line graphs may induce some readers to perceive x-axis variables as continuous even when they are in fact categorical (Zacks and Tversky, 1999). For this reason, bar graphs might be considered preferable for the stimuli of the present study because the x-axis variables were categorical. However, Kosslyn (2006) has argued, to the contrary, that even in such cases, line graphs are to be preferred over bar graphs when graph readers are expected to attend to the presence or absence of interaction effects. The reason is that interaction effects are associated with particularly salient visual effects in line graphs, such as the “x” shape produced by crossed lines or the sideway “v” shape discussed earlier, and therefore facilitate the identification of such effects relative to bar graphs. Thus, line graphs were preferred for the present study because detection of interaction effects constituted one of the three tasks participants were asked to perform.

Numeric data labels showing the exact value associated with each data point were included in the graphical stimuli for both test and training (e.g., Figure 1A). Although the inclusion of such labels is not standard in graph interpretation research, it has been shown to improve graph reading accuracy and speed (Calcaterra and Bennett, 2003). Numeric data labels were included in the present study to ensure that the graphs were informationally equivalent to the tables and text passages, so that any effects of format on task performance could not be due to the absence of precise numeric information in graphs. While this design decision would appear likely to decrease any differences in performance between graphs and tables, to anticipate our results, such differences were found nevertheless. It is expected that such differences would be as large or larger between tables and more typical graphs in which data labels are not included.

#### Procedure

The experiment was divided into 3 sections, i.e., one for each of the three effects described in the Materials: treatment, interaction, and secondary effect. The treatment effect section was always presented first, followed by the interaction effect section, with the secondary effect section always last. Each section consisted of a tutorial followed by a test. The tutorials and tests were administered through a computer interface.

The tutorials regarding treatment and interaction effects each provided instruction as to how to judge the presence or absence of the effect in question. In each of these tutorials, participants first read a description of the cognitive enhancer study used as the basis for all training examples. They were then provided with an explicit standard for how to judge whether the relevant effect was present or not, namely that the effect should be deemed present if a certain pair of numbers differed by a large amount, i.e., at least 5, and absent otherwise^2^. For the treatment effect section, the numbers in question were the marginal means for the two levels of the treatment factor, while for the interaction effect section, the numbers were the differences in means of the two levels of the treatment factor for the two levels of the secondary factor. Participants practiced applying this standard to the four training examples for the relevant effect, i.e., one graph and one table each for the two datasets illustrating the presence and absence of that effect, and received feedback on their responses. The examples were presented in pairs, with the details of which examples were paired together being varied according to a training manipulation which, because it had no significant effect on any of the measures discussed in the Results, is not described here. Details regarding the training manipulation are available in the Supplementary Materials.

The tutorial regarding secondary effects followed a similar procedure, except that participants were not provided with an explicit standard for judging whether a secondary effect was present, nor did they receive feedback on their responses. Because participants were not trained to perform the secondary task, this task constituted a stringent test of transfer which can be used to compare the different training conditions. In the absence of positive transfer due to training, participants were expected to perform worse on the secondary task compared to the other tasks, and in particular compared to the treatment task.

The test for each task was administered immediately following the corresponding tutorial. At the beginning of each test, participants were shown a description of the taste test study used as a basis for all test stimuli, and were told they would need to determine whether the given effect was present or absent in a series of possible results of the study. Each of the 48 test stimuli was shown once, in random order. Each stimulus remained on screen until participants indicated whether the given effect was present or absent using the mouse. No feedback was given.

The experiment may be viewed online at http://perceptsconcepts.psych.indiana.edu/experiments/dwb/MRIS_02/experiment_demo_live.html.

#### Measures

For each combination of task and format, accuracy was calculated as the percent of trials answered correctly. Note that participants completed 16 trials for each combination of task and format, i.e., one trial for each of the 16 datasets used to generate the test stimuli. Mean response time (RT) for each such combination was calculated as a second dependent measure.

### Results

#### Accuracy

Mean accuracy across tasks and formats was 68.8%, indicating that the task was challenging for participants. The accuracy data were submitted to a mixed logit model, using the *glmer* function from the *lme4* library for R (Bates et al., 2013). Mixed logit models have been recently recommended in preference to ANOVA applied to either raw or arcsine-square-root-transformed data resulting from binary forced-choice tasks (Jaeger, 2008), which may violate various assumptions of ANOVA.

A significant main effect of task on accuracy was found, χ^2^ (2) = 50.91, *p* < 0.001. Accuracy was highest for the treatment task (74.0%), intermediate for the interaction task (69.1%), and lowest for the secondary task (63.3%), for which participants did not receive explicit training. However, accuracy was significantly higher than chance (i.e., 50.0%) in all three cases, *p*s < 0.001.

**Figure 2:**
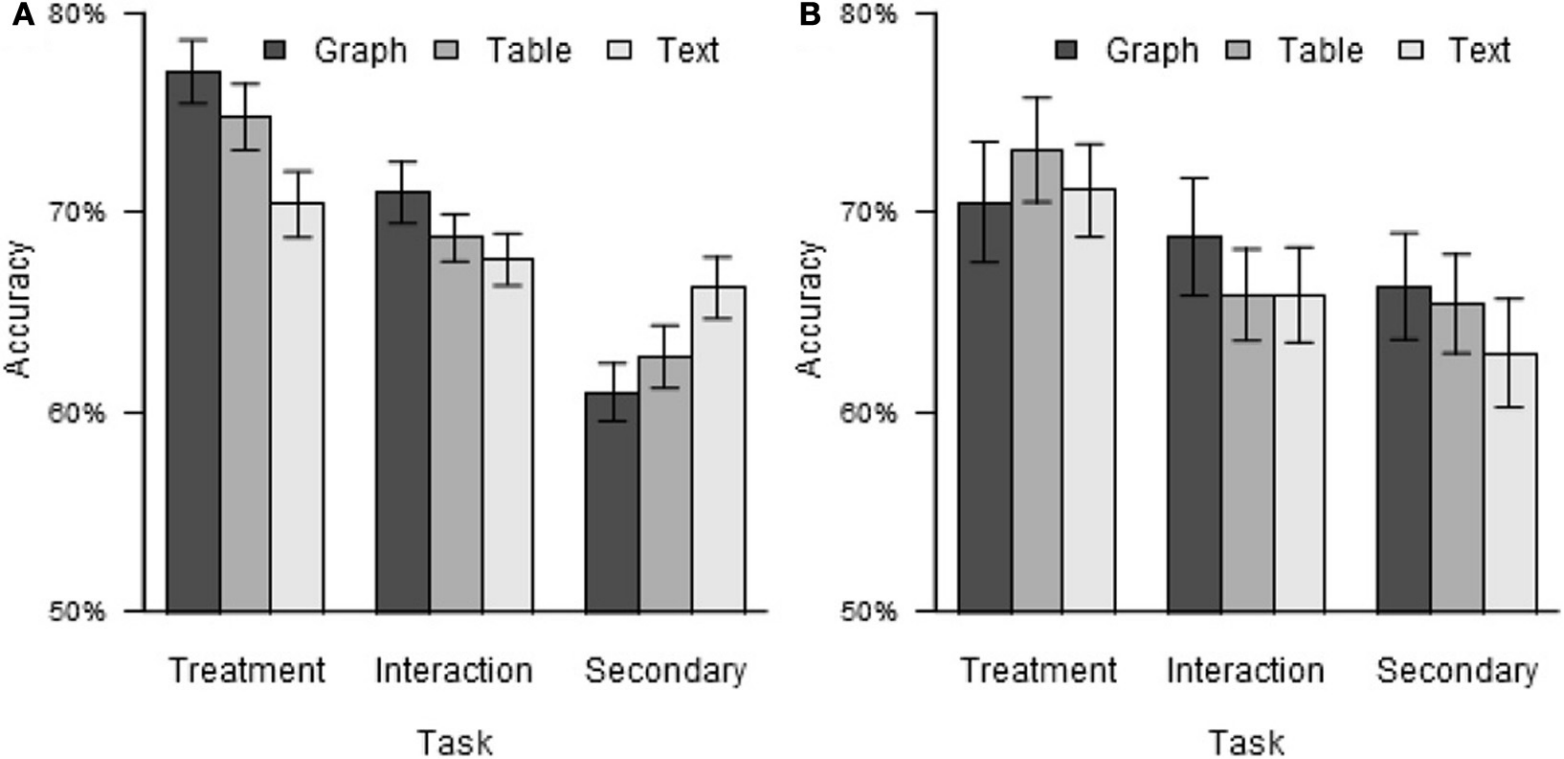
**Test accuracy by task and format. (A)** Experiment 1. **(B)** Experiment 2. Error bars indicate standard errors, here and elsewhere.

While the main effect of format did not reach significance, χ^2^ (2) = 5.75, *p* = 0.056, its interaction with task did, χ^2^ (4) = 55.15, *p* < 0.001. The data relevant to this interaction are shown in Figure 2A. Because some of the research questions relate to the comparison between graphs and tables and focus on the comparison between the treatment and secondary tasks, the above analysis was repeated with the data for text stimuli and for the interaction task excluded. The interaction of format with task was still significant, χ^2^ (1) = 7.57, *p* = 0.006. As shown in Figure 2A, accuracy was highest for the treatment task, intermediate for the interaction task, and lowest for the secondary task for both graphs and tables, but the difference in accuracy between the treatment and secondary tasks was greater for graphs (treatment task: 77.0%, secondary task: 61.0%, difference: 16.0%) than for tables (treatment task: 74.8%, secondary task: 62.7%, difference: 12.0%). No other significant effects on accuracy were found.

#### Response time

For analysis of RT, for each participant, trials with RT differing from that participant's mean RT by more than 2 standard deviations were discarded. In the remaining data, the mean RT was 6.00 s, and ranged from 1.59 to 18.14 s. The RT data were analyzed using the same ANOVA model structure as was used for the accuracy data. As in the case of accuracy, a significant main effect of task on RT was found, *F*(2, 248) = 56.28, *p* < 0.001, η^2^_G_ = 0.078. Participants were slowest on the treatment task (7.48 s), intermediate on the interaction task (5.69 s), and fastest on the secondary task (4.84 s).

**Figure 3:**
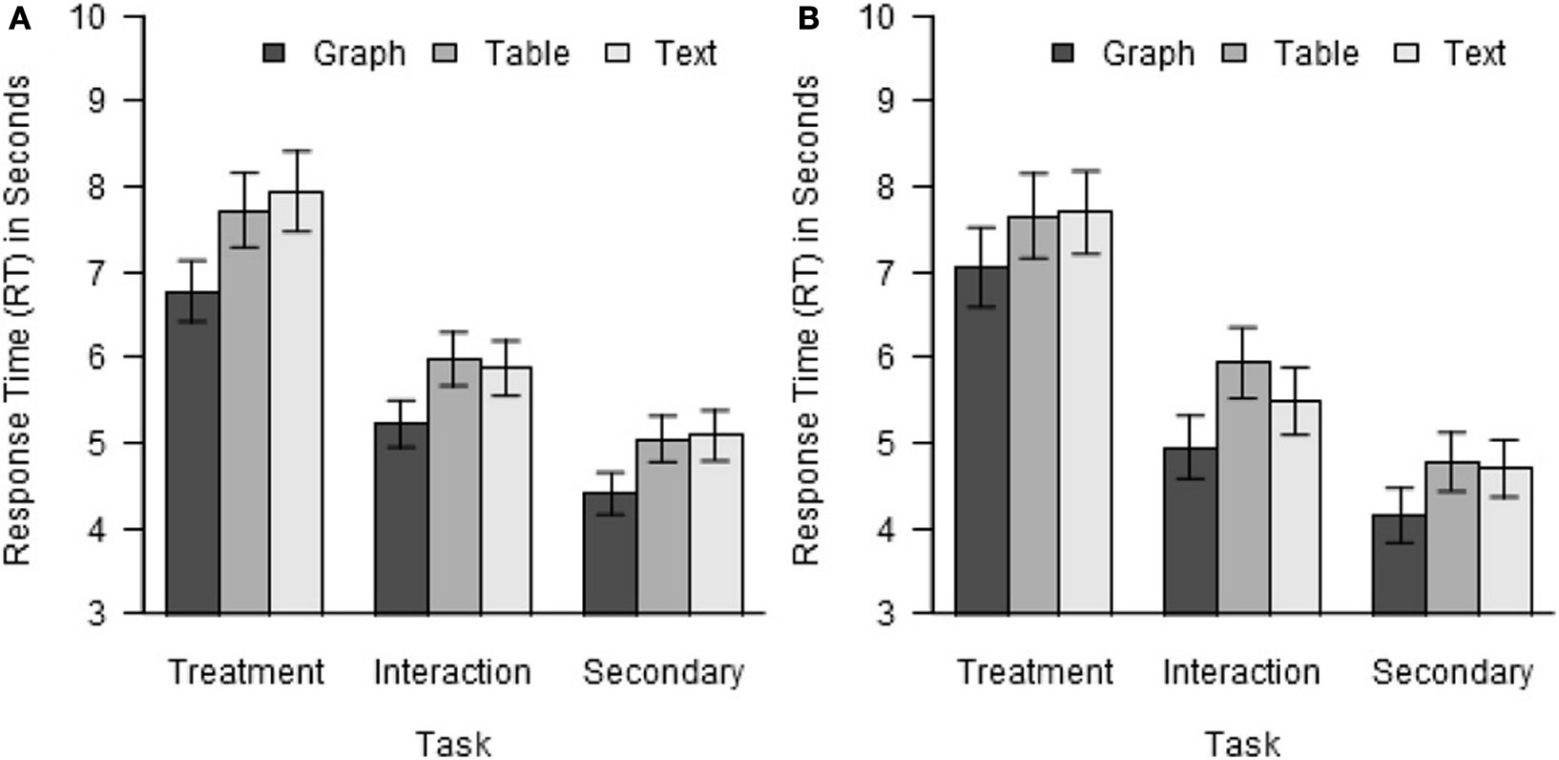
**Response time by task and format. (A)** Experiment 1. **(B)** Experiment 2.

More important to the objectives of the research, the main effect of format was also significant, *F*(2, 248) = 37.96, *p* < 0.001, η^2^_G_ = 0.010. Participants performed the tasks reliably faster with graphs (5.46 s) than with tables (6.25 s) or text (6.29 s). This main effect was qualified by a significant interaction of format with task, *F*(2, 248) = 37.96, *p* = 0.029, η^2^_G_ < 0.001. As shown in Figure 3A, while all three tasks were performed faster with graphs than with tables or text, but the difference was largest for the treatment task and smallest for the secondary task. As for accuracy, the above analysis was repeated with the data for text stimuli and interaction effects excluded. The main effect of format remained significant, *F*(1, 124) = 58.55, *p* < 0.001, η^2^_G_ = 0.012, but the interaction of format with task did not, *F*(1, 124) = 3.59, *p* = 0.060. No other significant effects on RT were found.

#### Influence

As described in the Introduction, a possible factor influencing flexibility of representational formats is intrinsic bias, and one possible cause of intrinsic bias is differences in the salience of the representational properties relevant to detecting different effects. This possibility led to the prediction that, if intrinsic bias is present and caused by such differences in salience, the more salient representational properties could influence responses even in tasks for which they are irrelevant. Additional analyses were conducted to test this prediction.

Because the specific representational properties on which participants relied to perform the tasks are not known, our analysis of influence is conducted at the level of effects, essentially treating each effect as a single feature corresponding to the presence or absence *en masse* of all properties relevant to that effect. Influence *I_x,t_* from each effect *x* on each task *t* was calculated using the following formula:

$$I_{x,t}=P\left(r|x,t\right)-P(r|\sim x,t)$$

*P*(*r*|*t*) signifies the probability of a positive response, indicating that the effect being queried is present, when the present task is *t*. In other words, *P*(*r*|*t*) is the probability of hits plus the probability of false alarms. *P*(*r*|*x, t*) and *P*(*r*|~ *x, t*) signify, respectively, the probabilities of positive response on task *t* when effect *x* is present and when effect *x* is absent. Thus, *I_x,t_* represents the degree to which the presence of effect *x* increases the probability of positive response on task *t*.

Because *P*(*r*|*x, t*) and *P*(*r*|~ *x, t*) each may range from 0 to 100%, and are in principle independent of each other, *I_x,t_* may range from −100% to 100%. When *x* = *t*, i.e., effect *x* is the effect relevant to task *t*, perfect responding would yield *P*(*r*|*x, t*) = 100% and *P*(*r*|~ *x, t*) = 0%, resulting in *I_x,t_* = 100%, while random responding would yield *P*(*r*|*x, t*) = 50% and *P*(*r*|~ *x, t*) = 50%, resulting in *I_x,t_* = 0%. Thus, for *x* = *t*, values of *I_x,t_* greater than 0% indicate positive influence of the effect *x* on the task to which it is relevant. On the other hand, when *x* ≠ *t*, i.e., effect *x* is irrelevant to task *t*, perfect responding would yield *P*(*r*|*x, t*) = 50% and *P*(*r*|~ *x, t*) = 50%, resulting in *I_x,t_* = 0%. Random responding would also yield *I_x,t_* = 0%, for the same reason given above. Thus, for *x* ≠ *t*, values of *I_x,t_* other than 0% would indicate that responses for task *t* were influenced by the irrelevant effect *x*.

**Figure 4:**
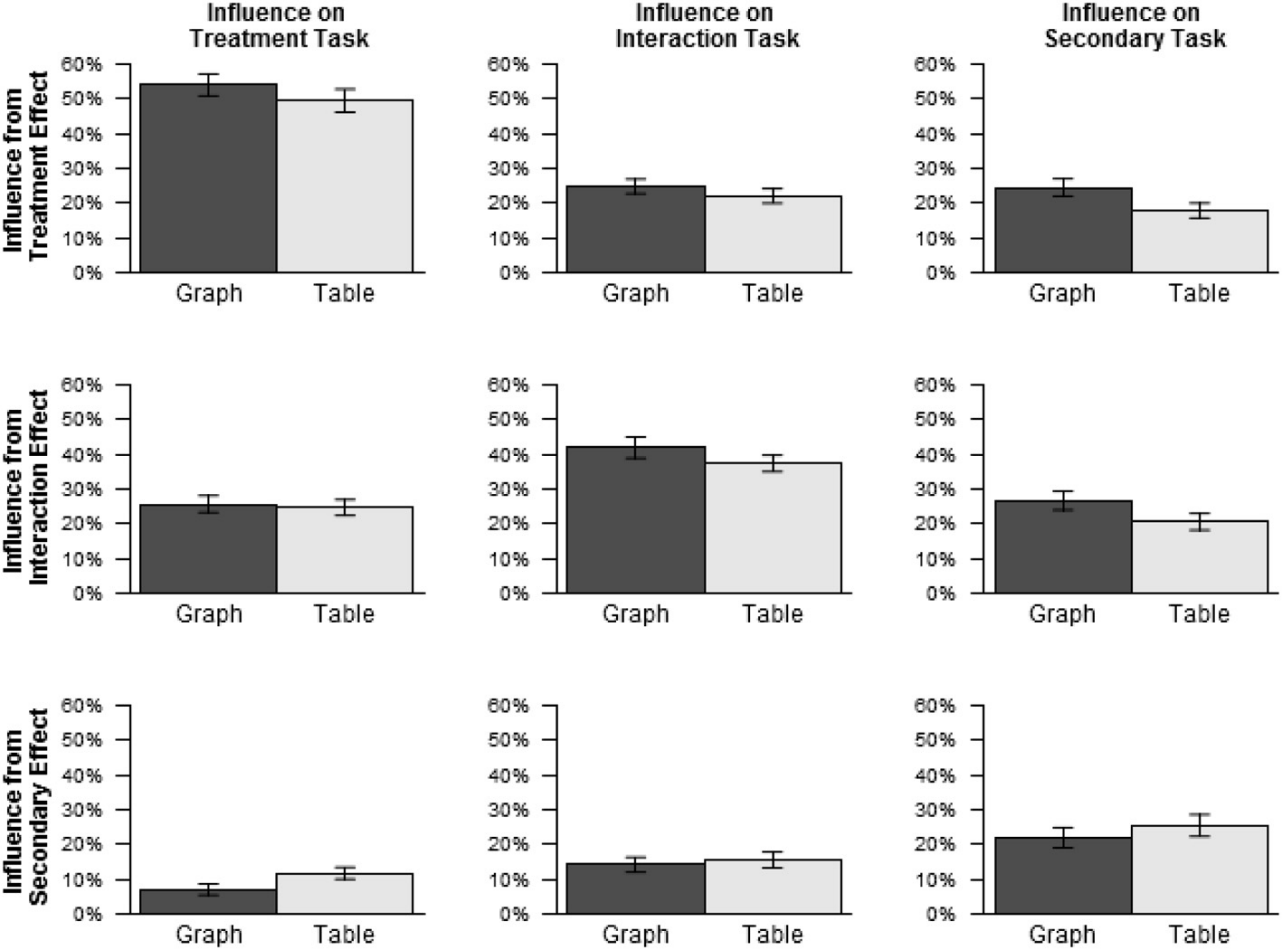
**Influence by influencing effect and influenced task for graphs and tables (Experiment 1)**.

Figure 4 shows the average values of influence *I_x,t_* from each influencing effect *x* on each influenced task *t*, excluding the data for text stimuli, as the possibility of intrinsic bias was mainly of interest with respect to graphs and tables. Influence was greater than 0% for all combinations of influencing effect and influenced task, *p*s < 0.001, including, in particular, all cases of irrelevant influence, shown in the off-diagonal cells of Figure 4. Thus, responses were influenced by irrelevant effects more than either perfect responding or random guessing would predict.

For graphs, such irrelevant influence increased from the earlier tasks to the later tasks. Irrelevant influence was 16.3% on the treatment task, 19.5% on the interaction task, and 25.6% on the secondary task. By contrast, for tables, irrelevant influence was about the same for all three tasks: treatment: 18.2%, interaction: 18.8%, secondary: 19.2%. Paired Wilcoxon signed ranks tests were conducted for each pair of tasks separately for graphs and tables, using a Bonferroni correction for multiple comparisons. For graphs, irrelevant influence was greater on the secondary task than on the treatment task, *p* < 0.001, and marginally greater on the secondary task than on the interaction task, *p* = 0.099. No other differences were significant, *p*s > 0.95. In sum, while accuracy decreased more across tasks with graphs than with tables, irrelevant influence increased across tasks with graphs, and did not do so with tables. This result is consistent with the possibility that, for graphs, the representational properties relevant to detecting treatment and interaction effects were more salient than those relevant to detecting secondary effects, while for tables, no such difference existed^3^.

For both influence and accuracy, the effect of task was greater for graphs than for tables. The similarity of these results suggests the possibility of a statistical association between influence and accuracy. To test for this possibility, for each participant, average influence from irrelevant effects was calculated for each combination of task and format, and influence score was then added as a predictor to the mixed logistic regression model used to analyze the accuracy scores. (The data for text stimuli were again excluded.) This analysis found a significant negative effect of irrelevant influence on accuracy, χ^2^ (1) = 105.39, *p* < 0.001, indicating that accuracy tended to be lower when irrelevant influence was higher. Importantly, this negative effect is not necessitated by the way influence was calculated. For example, random guessing would lead both to low influence and low accuracy.

### Discussion

Across three data interpretation tasks requiring attention to relationships among data points, accuracy did not vary significantly with representational format, while RTs were significantly faster with graphs than with tables. Faster performance without loss of accuracy suggests that graphs induce more efficient processing than tables for the present tasks. This result is consistent with advantages found in other studies of graphical over tabular format for tasks involving relationships among data points (Vessey and Galletta, 1991; Meyer et al., 1999; Porat et al., 2009; Schonlau and Peters, 2012).

In addition to this main effect of format, a significant interaction of format with task was found with respect to accuracy. The difference in performance across tasks was greater with graphs than with tables, suggesting lower flexibility with graphs. The intuition that graphs can be used to favor certain perspectives for data interpretation over others is common, and implies that graphs may not support flexible switching between different perspectives, as required between the treatment and secondary tasks, for example. To our knowledge, the present results are novel in providing experimental evidence for this intuition of low flexibility, and for demonstrating that tables exhibit relatively higher flexibility by the same standard. Consistent with this result, graphs, but not tables, showed differences in irrelevant influence across tasks, suggesting that certain visual features of graphs may be highly salient and, furthermore, participants may have had difficulty ignoring these salient features when they were irrelevant to the task at hand.

However, the results of Experiment 1 are ambiguous in two respects. First, as discussed in the Introduction, low flexibility might result from at least two different causes: intrinsic bias, in which some tasks are intrinsically easier than others to perform with a given representation, and transfer of practice, in which experience with one task has a negative effect on performance with a subsequent task. Thus, the format * task interaction could result either from greater intrinsic bias or from greater (negative) transfer of practice for graphs, compared to tables. The design of Experiment 1 does not permit disambiguation between these possibilities because the order of tasks was fixed. For example, the secondary task was always encountered last, so low performance on the secondary task could be due either to negative transfer of practice from the preceding tasks, or to intrinsic bias favoring the preceding tasks. Experiment 2 was designed to address this issue.

If the format * task interaction is assumed to result from intrinsic bias, the results are ambiguous in another respect. Specifically, because participants were trained on the treatment task but not on the secondary task, better performance on the former would be expected even without an effect of format. As a result, intrinsic bias due to representational format could have caused the observed format * task interaction in two different ways. First, graphs could facilitate detection of effects of the legend variable, relative to the x-axis factor. Because the legend always represented the treatment factor, such facilitation would have increased accuracy on the treatment task, adding to the performance improvement expected due to training, and thus increasing the difference in accuracy between that task and the secondary task. Alternatively, tables could facilitate comparison between columns, relative to comparison between rows. Because the columns always represented levels of the secondary factor, such facilitation would have increased accuracy on the secondary task, partially counteracting the performance decrement due to the absence of training, and thus decreasing the difference in accuracy between that task and the treatment task. Thus, the observed format * task interaction could have been caused either by intrinsic bias for graphs favoring the legend variable, or by intrinsic bias for tables favoring column comparison. While either of these explanations are compatible with the results regarding accuracy, the first possibility is supported by the analysis of irrelevant influence, which found larger effects of task, suggesting larger differences in the saliences of relevant stimulus properties, with graphs than with tables. This issue was further addressed in Experiment 2.

## Experiment 2

Experiment 2 employed largely the same methodology as Experiment 1, with one crucial change. During the generation of graphs and tables as training examples and test stimuli, the visual dimensions previously assigned to treatment factors were re-assigned to secondary factors, and vice versa. That is, for graphs (Figure 1C), the legend now represented secondary factors and the x-axis treatment factors, while for tables (Figure 1D), the rows now represented secondary factors and the columns treatment factors. However, the tutorial and test for the treatment task were still administered first, and those for the secondary task last. The different possible explanations of the results of Experiment 1 lead to different predictions for how the changes made in Experiment 2 would affect those results.

First, if the format * task interaction found in Experiment 1 was due to transfer of practice effects, then the same interaction should appear in Experiment 2, because the temporal sequence of tasks allowed transfer effects for the same tasks, i.e., the interaction and, especially, secondary tasks, in both experiments. By contrast, if the interaction was due to greater intrinsic bias in one or the other representational format favoring visual features relevant to some tasks more than those relevant to others, then that interaction should be significantly changed in Experiment 2 due to the alteration in the visual features relevant to each task. In particular, for the treatment and secondary tasks, a reversal of the interaction would be expected, because the visual dimensions relevant to each of these tasks in Experiment 1 were precisely reversed in Experiment 2.

Second, two different explanations involving intrinsic bias were mentioned earlier. These explanations also lead to different predictions regarding Experiment 2. If the results of Experiment 1 were due to intrinsic bias favoring comparison between columns with tables, then accuracy with tables in Experiment 2 should improve for the treatment task and worsen for the secondary task, because treatment factors are represented by table columns, but secondary factors by table rows, in Experiment 2. On the other hand, if the results of Experiment 1 were due to intrinsic bias favoring detection of main effects of the legend variable with graphs, then accuracy with graphs in Experiment 2 should worsen for the treatment task and improve for the secondary task, because treatment factors were represented by the x-axis, but secondary factors by the graph legend, in Experiment 2.

Importantly, the latter version of the intrinsic bias explanation, which assumes that graphs are biased in favor of detecting main effects of the legend variable, does not necessarily predict better performance on the secondary task than on the treatment task in Experiment 2. Although, according to this explanation, representation of secondary factors in the graph legend should facilitate performance on the secondary task relative to the treatment task, any such facilitation could still be outweighed by participants' receiving training on the treatment task and not on the secondary task. Also, even the presence of negative transfer from the treatment to the secondary task in Experiment 2 would still be compatible with the intrinsic bias explanation, which does not deny the possibility of such negative transfer, but rather denies that the format * task interaction found in Experiment 1 was due to format-related differences in the degree of negative transfer.

### Methods

The method employed in Experiment 2 was the same as that employed in Experiment 1 in all respects except those mentioned below.

#### Participants

Participants were *N* = 43 undergraduate students from the Indiana University Psychology Department who participated in partial fulfillment of a course requirement. Importantly, all participants were drawn from the same source as in Experiment 1, suggesting that systematic differences in samples between the two experiments are unlikely. Participants were approximately evenly split between males (*N* = 19) and females (*N* = 24). Most (*N* = 39) participants were aged 18–21, with the remainder (*N* = 4) aged 22 or older. The majority of participants (*N* = 31) reported never having learned about main and interaction effects before the experiment, and as reported previous experience had no effect on test accuracy, *p* = 0.882, it was not included as a factor in the subsequent analyses.

#### Materials

The test and training stimuli were based on those used in Experiment 1. For each of the graphical and tabular stimuli used in Experiment 1 (Figures 1A,B), a modified version was created by reversing the assignments of the x-axes/legends of graphs (Figure 1C) or the columns/rows of tables (Figure 1D) to the treatment and secondary factors. That is, while in Experiment 1, the legends of graphs and the rows of tables always represented the treatment factor, in Experiment 2, they represented the secondary factor. Similarly, while in Experiment 1, the x-axes of graphs and the columns of tables represented the secondary factor, in Experiment 2, they represented the treatment factor. The stimuli for Experiment 2 were generated using the same datasets as in Experiment 1 and were in all other respects the same as those used in Experiment 1. In contrast to the graphical and tabular stimuli, the text passage stimuli were not changed, i.e., they were exactly the same as those used in Experiment 1 (Figure 1E).

#### Procedure

The same procedure was used in Experiment 2 as in Experiment 1, with the exception that the explanations of how to determine the presence of treatment and interaction effects were modified to take account for the changes made to the stimuli. For example, references to table rows were changed to refer to table columns.

### Results

#### Accuracy

Mean accuracy across tasks and formats was 67.4%, comparable to that observed in Experiment 1 (68.8%). While accuracy across tasks showed the same trend as in Experiment 1, i.e., decreasing performance from the first to the last task (treatment task: 71.6%, interaction task: 66.8%, secondary task: 64.9%), the magnitude of this trend was reduced relative to Experiment 1. Nevertheless, analysis of the accuracy data using the same mixed logit model structure as in Experiment 1 still found a reliable effect of task, χ^2^ (2) = 7.90, *p* = 0.019. No other significant effects or interactions were found, *p*s > 0.10.

The accuracy data by task and format are shown in Figure 2B. Contrary to the prediction of the transfer of practice explanation for the results of Experiment 1, but consistent with that of the intrinsic bias explanation, the qualitative pattern of the format * task interaction was reversed in Experiment 2, although the interaction was not statistically reliable in Experiment 2 alone. For example, the difference in performance between the treatment and secondary tasks was greater for graphs than for tables in Experiment 1, but was slightly greater for tables (treatment task: 73.1%, secondary task: 65.4%, difference: 7.7%) than for graphs (treatment task: 70.5%, secondary task: 66.3%, difference: 4.2%) in Experiment 2.

The intrinsic bias explanation of Experiment 1 allowed for two variations. The first variation involved bias for graphs favoring detection of effects of the legend variable, and predicted poorer performance on the treatment task and improved performance on the secondary task with graphs in Experiment 2, while the second variation involved bias for tables favoring comparison between columns, and predicted improved performance on the treatment task and poorer performance on the secondary task with tables in Experiment 2. In fact, accuracy on the treatment task was poorer for both graphs (Experiment 1: 77.0%, Experiment 2: 70.5%) and tables (Experiment 1: 74.8%, Experiment 2: 73.1%), while accuracy on the secondary task was improved for both graphs (Experiment 1: 61.0%, Experiment 2: 66.3%) and tables (Experiment 1: 62.7%, Experiment 2: 65.4%). Evidently, this result is consistent with the explanation involving greater intrinsic bias for graphs favoring detection of effects of the legend variable, and not with that involving greater intrinsic bias for tables favoring comparison between columns.

The data from both experiments were pooled in order to test the reliability of the differences between their results. Although the possibility of systematic differences between the experiments, such as differences in the sample populations, cannot be ruled out, the likelihood of such differences is reduced by the similarity in methodology between experiments and their reliance on the same source for experimental participants. Analysis of the pooled data, with experiment number added as a between-subjects factor, found significant effects of task, format, and their interaction, qualified by a significant three-way interaction of experiment, task, and format, χ^2^ (4) = 17.55, *p* = 0.002, suggesting that the reversal between experiments of the task * format interaction was reliable. To better understand this interaction, the data for graphs and tables were analyzed separately without format as a factor. The interaction of experiment and task was significant for graphs, χ^2^ (2) = 11.14, *p* = 0.004, indicating that the changes in performance for the treatment and secondary tasks with graphs were reliable, supporting the first of the two intrinsic bias accounts described above. The interaction of experiment and task was not significant for tables, χ^2^ (2) = 2.71, *p* = 0.258, providing no support for the second of the two intrinsic bias accounts (and as described above, the qualitative trend of the data was opposite to that predicted by the second account). All of the results just described were qualitatively unchanged when the data for text stimuli and interaction effects were excluded.

#### Response time

The RT data were filtered in the same way, and analyzed using the same model, as in Experiment 1. Average RT was 5.82 s, and ranged from 1.20 to 16.3 s. As in Experiment 1, significant effects of task and format were found, *F*(2, 80) = 38.24, *p* < 0.001, η^2^_G_ = 0.179 for task and *F*(2, 80) = 14.08, *p* < 0.001, η^2^_G_ = 0.014 for format. As shown in Figure 3B, participants were slowest on the treatment task and fastest on the secondary task, and were faster with graphs than with tables for all three tasks. In contrast to Experiment 2, the interaction of format with task was not significant, *F*(4, 160) = 0.82, *p* = 0.514, nor were any other significant effects found, *p*s > 0.05.

#### Influence

**Figure 5:**
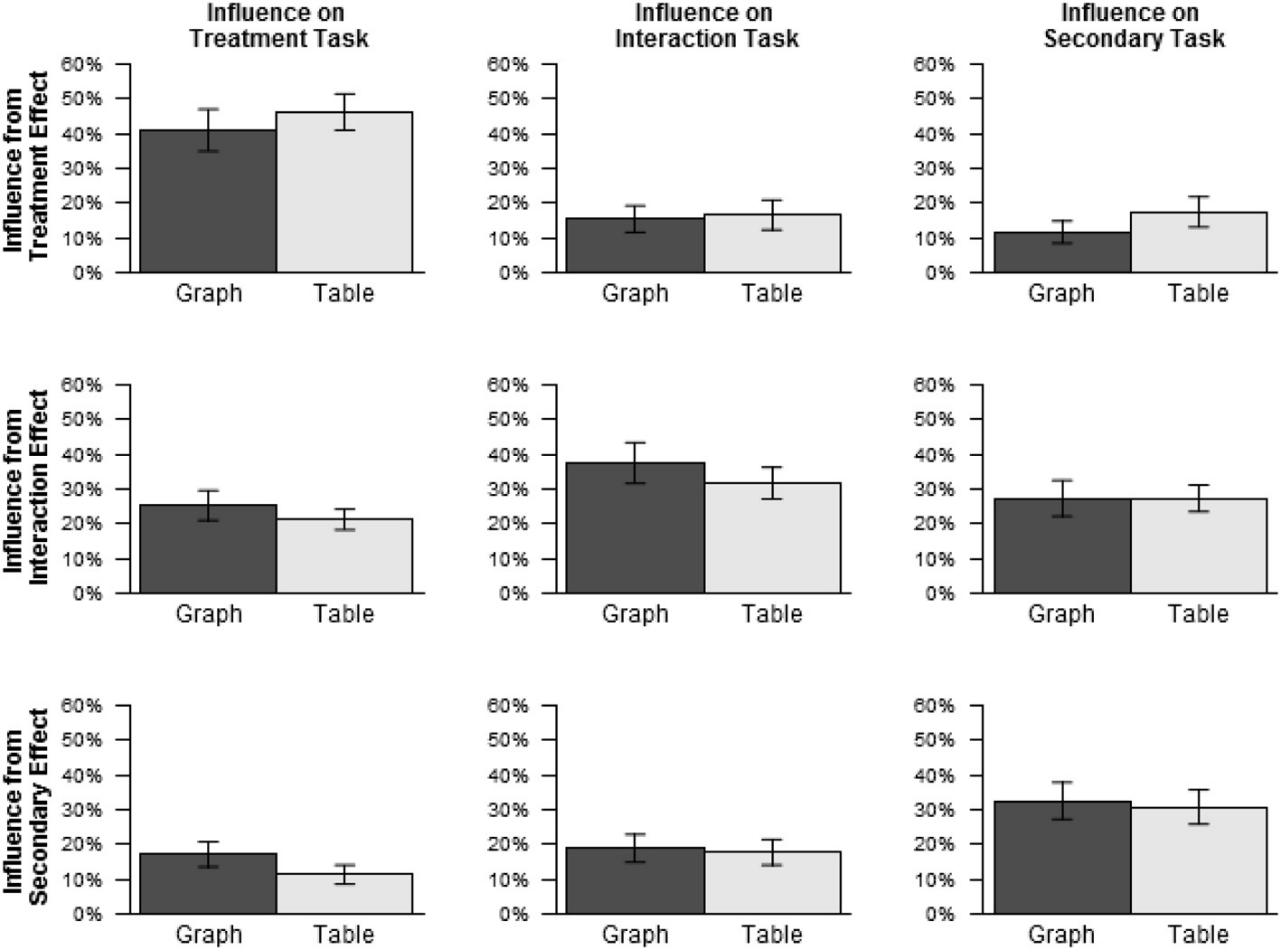
**Influence by influencing effect and influenced task for graphs and tables (Experiment 2)**.

Influence *I_x,t_* was calculated in the same manner as in Experiment 1. Figure 5 shows average influence from each effect on each task, excluding data for text stimuli. As in Experiment 1, influence was greater than 0% for all combinations of influencing effect and influenced task, *p*s < 0.001, including in particular all cases of irrelevant influence, shown in the off-diagonal cells of Figure 5. Influence from irrelevant effects decreased slightly across tasks with graphs (treatment: 21.2%, interaction: 17.2%, secondary: 19.5%) and increased slightly across tasks with tables (treatment: 16.3%, interaction: 17.2%, secondary: 22.4%), showing the opposite trend as in Experiment 1. However, paired Wilcoxon signed ranks tests comparing irrelevant influence between tasks for each pair of tasks separately for graphs and tables found no significant differences, *p*s > 0.80.

To ascertain whether the negative association between influence from irrelevant effects and accuracy found in Experiment 1 was replicated in Experiment 2, irrelevant influence was added as a predictor to the model used to analyze the accuracy scores, as in Experiment 1. Just as in Experiment 1, this analysis found a significant negative effect of irrelevant influence on accuracy, χ^2^ (1) = 41.22, *p* < 0.001, indicating that accuracy tended to be lower when irrelevant influence was higher.

### Discussion

The results of Experiment 2 serve to disambiguate between several possible explanations for those of Experiment 1. First, the reversal between experiments of the format * task interaction effect on accuracy confirms the predictions of the intrinsic bias explanation and disconfirms those of the transfer of practice explanation. Although negative transfer of practice may have occurred, as evidenced by the decrease in accuracy from the treatment to the secondary task in both experiments, the assumption that one representational format led to more negative transfer than the other cannot account for the reversal of the format * task interaction observed in Experiment 2. Second, the fact that the reversed assignments of visual dimensions to factors used in the stimuli of Experiment 2 resulted in lower accuracy on the treatment task and higher accuracy on the secondary task for graphs confirms the predictions of the intrinsic bias account involving an advantage for detecting effects of the legend variable with graphs, while the fact that the opposite trends were *not* observed for tables disconfirms that predictions of the intrinsic bias account involving an advantage for comparison between columns with tables. In summary, the results of Experiment 2 support an interpretation of the format * task interaction in Experiment 1 as due to lower flexibility for graphs, and in particular, to greater intrinsic bias for graphs, favoring comparison of levels of the legend variable rather than of the x-axis factor.

It is worth noting that for both the treatment and interaction tasks, the difference in performance between experiments was greater for graphs than for tables. For example, performance on the treatment task was much better in Experiment 1 than in Experiment 2 for graphs (77.0 vs. 70.5%), but only slightly better for tables (74.8 vs. 73.1%). This comparison relates to the second possible operationalization of flexibility mentioned in the Introduction, i.e., differences in performance on a single task depending on assignment of visual dimensions to factors. The fact that graphs also showed greater performance differences than tables on this measure supports the conclusion that graphs are a less flexible representational format.

In Experiment 2, as in Experiment 1, faster RTs were accompanied by lower accuracy across the three data interpretation tasks. These results may be partially explained in terms of a speed-accuracy tradeoff favoring speed over accuracy to an increasing degree over the course of the experiment. For example, participants may have experienced fatigue as the experiments proceeded, leading to faster but less accurate responses on the tasks presented later. However, the reversal of the format * task interaction for accuracy in Experiment 2 cannot be explained by such a speed-accuracy tradeoff, because no similar reversal of the format * task interaction was observed for RT. In fact, the pattern of RT data was quite similar between experiments, with RTs consistently faster with graphs than with tables, both for tasks on which accuracy was higher for graphs and for tasks on which accuracy was lower for graphs. A possible explanation is that, compared to tables, graphs induce a sense of fluency (Oppenheimer, 2008) which does not depend entirely on actual competence with the task and results in fast reaction times, while compared to graphs, tables require effortful processing even when the task is well-understood, leading to slower reaction times.

## General discussion

The present findings support the conclusions of previous research that graphs are, overall, preferable to tables for the performance of tasks involving relationships among data points. In both experiments, participants detected statistical effects as accurately, and more quickly, with graphs than with tables, indicating that graphs induce more efficient performance of this type of task. This result is consistent with the view, presented in the Introduction that graphs permit efficient processing of relationships among data points because such relationships correspond to visual patterns which may be encoded as single features (Pinker, 1990; Pomerantz and Portillo, 2012), while tables do not afford such encoding shortcuts.

The principal new finding of the present study is that graphs, while permitting more efficient task performance overall, may also afford less flexibility in data interpretation than do tables. That is, compared to tables, there is a relatively large difference in the accuracy with which interpretive tasks of comparable difficulty are performed with graphs. These findings dovetail with previous findings, obtained using free-response graph interpretation paradigms, suggesting differences in the types and frequencies of interpretations associated with variables represented by different visual dimensions in graphs of bivariate data (Shah and Carpenter, 1995; Carpenter and Shah, 1998; Shah et al., 1999; Shah and Freedman, 2011). The present results are novel in demonstrating that asymmetries also appear in accuracy scores from a forced-choice paradigm using predefined interpretive tasks, i.e., detection of specific types of statistical effects. Another novel contribution of the present study is the finding that such asymmetries are reduced when the same tasks are performed using tables of the same data.

In addition to demonstrating lower flexibility across tasks performed with graphs, compared to tables, the present study offers some evidence to clarify the cause for such reduced flexibility. Two possible causes were considered: negative transfer of practice, in which experience with earlier tasks negatively impacted performance on later tasks, and intrinsic bias, in which performance differences result from differences in the intrinsic ease of performing the tasks. The results of Experiment 2 were consistent with the intrinsic bias account and not with the transfer of practice account.

Importantly, the selection of the intrinsic bias account in preference to the transfer of practice account for the format-related differences in flexibility found in Experiment 1 does not imply that previous experience played no role in producing those differences. Rather, it implies only that those differences were not produced by differing effects of experience with the experimental tasks. Indeed, the observed bias may well have been a result of experience with graphs and/or tables prior to the experiment, and different experiences could have reduced, increased, or even reversed such bias. The extent to which such bias is consistent or, alternatively, varies across individuals with different experiences of graph and table reading could be an interesting topic for future study.

The Introduction described one mechanism which might give rise to intrinsic bias. In the proposed mechanism, differences in the salience of stimulus properties relevant to different tasks could lead to facilitation of some tasks, whose relevant stimulus properties were highly salient, relative to other tasks, whose relevant stimulus properties were less salient. This possibility led to the prediction that the presence or absence of properties relevant to the facilitated task(s) would influence responses on the other tasks, despite being irrelevant to the latter. This prediction was confirmed. In Experiment 1, influence from irrelevant effects was highest precisely when performance was lowest, i.e., with graphs on the secondary task. Also, in both experiments, higher influence from irrelevant effects was strongly associated with lower accuracy, consistent with the possibility that lower accuracy was caused in part by interference from salient stimulus properties associated with irrelevant effects.

Task responses for both graphs and tables showed a high degree of influence from irrelevant effects, suggesting that such influence was not caused entirely by stimulus properties unique to graphs. However, the *difference* between tasks in the degree of such influence observed for graphs, but not for tables, is presumably due to stimulus properties unique to graphs. Moreover, the evidence that graphs induced a bias to attend specifically to effects of the legend variable, as discussed below, suggests that the more salient visual properties in question are among those associated with such effects, e.g., differences in line height as opposed to, for example, differences in line slope, as well as specific visual configurations such as the sideways “v” shape in Figure 1A. However, determining specifically which of these properties are primarily responsible for the observed effects is beyond the scope of the present study. While recognition and interpretation of visual features play an important role in models of graph comprehension (Pinker, 1990; Carpenter and Shah, 1998; Freedman and Shah, 2002; Trickett and Trafton, 2006; Ratwani et al., 2008), little is known about specifically what type of visual features constitute the primary inputs to interpretive processes. This question could provide a fruitful direction for future research.

### Direction of intrinsic bias

The specific pattern of results in Experiments 1 and 2 clarifies the direction of the intrinsic bias induced by graphs of bivariate data. Importantly, the direction of such biases may differ by graph type (Shah et al., 1999; Shah and Freedman, 2011), so the following discussion may apply only to the type of graph employed in the present study, i.e., line graphs. For the tasks requiring recognition of main effects, i.e., the treatment and secondary tasks, accuracy was higher when the relevant variable was represented in the legend rather than in the x-axis. That is, accuracy for the treatment task was higher in Experiment 1 than in Experiment 2, while accuracy for the secondary task was higher in Experiment 2 than in Experiment 1. The reliability of these differences was supported by a significant experiment * task interaction in the accuracy data for graphical stimuli. Thus, accurately detecting main effects of the legend variable appears to be easier than detecting main effects of the x-axis variable.

Consistent with these findings, Shah and Freedman (2011) found that spontaneous interpretations of bivariate line graphs related more often to main effects of the legend variable than to main effects of the x-axis variable. However, other studies by Shah and her colleagues point in the opposite direction (Shah and Carpenter, 1995; Carpenter and Shah, 1998; Shah et al., 1999). Specifically, Shah and Carpenter (1995; see also Carpenter and Shah, 1998) found that metric properties of variables were more salient when variables were displayed on the x-axis rather than in the legend, while Shah et al. (1999) found that quantitative trends were likely to be noticed when associated with the x-axis rather than with the legend variable of bivariate line graphs. In light of these earlier results, Shah and Freedman (2011) proposed that their finding that interpretations involving main effects were more often related to the legend variable may have been due to a kind of response competition, in which statements regarding main effects of the x-axis variable were rare because effects of that variable were more often stated as interactions rather than as main effects. This proposal suggests that effects of the x-axis variable are in fact more salient than those of the legend variable, despite Shah and Freedman's (2011) apparent result to the contrary.

An analogous account for the present study's finding of higher accuracy when detecting main effects of the legend variable rather than the x-axis variable would hold that involuntary attention to interaction effects interfered with the tasks involving main effects of the x-axis variable, causing lower accuracy on those tasks, in the same way that competition from responses involving interaction effects may have reduced responses involving x-axis main effects in Shah and Freedman's (2011) study. However, for this account to explain the present findings requires that such interference was absent, or reduced, for the tasks involving main effects of the legend variable, because otherwise it could not have caused the observed difference in accuracy between the two types of task. Contrary to such an account, influence *I_x,t_* from interaction effects on the two main effect tasks did not differ between the two main effect tasks in either experiment^4^, suggesting that differences in performance between the two main effect tasks are unlikely to have resulted from differential interference from involuntary attention to interaction effects.

An alternative account involves differences in the types of variables and tasks best afforded by the legends and x-axes of line graphs. Graph readers are likely to perceive separate graph lines as units, consistent with the Gestalt principle of connectivity (Shah et al., 1999; see also Kessell and Tversky, 2011; Tversky et al., 2013). Perceiving graph lines as units could facilitate comparison between them, and this facilitatory effect would improve detection of main effects of the legend variable, consistent with the findings of the present study. However, graph readers' experience with function graphs may also create an expectation that the x-axis represents a continuous variable and each graph line depicts a function of that variable. When the x-axis *does* represent a continuous variable, as in the stimuli of Shah and her colleagues (Shah and Carpenter, 1995; Carpenter and Shah, 1998; Shah et al., 1999), these expectations would presumably draw attention to that variable's metric properties, as found by Shah and Carpenter (1995; Carpenter and Shah, 1998), and also increase the salience of functional relationships between the x-axis and y-axis variables, as found by Shah et al. (1999). These effects may not occur when the predictor variable is categorical rather than continuous, as in the stimuli of the present study. In summary, the effects of continuous predictor variables may be most salient when those predictors are represented on the x-axis, but the effects of categorical predictors may be more salient when those variables are represented in the graph legend.

### Implications for data representation

The present findings have practical implications for the use of graphs and tables for exploring and communicating data. By itself, the finding of greater efficiency in task performance with graphs compared to tables suggests that graphs should be preferred for tasks like those employed in the present study. However, such an implication is qualified by the finding that graphs are less flexible than tables, supporting relatively good performance on some tasks at the expense of others. This result suggests that whether graphs or tables are to be preferred for practical applications may depend not only on what tasks are involved, but also on whether flexibility across a range of different tasks is required. If data is to be represented with a specific perspective in mind, as is often the case when the primary goal is communication in support of a particular analysis, external representations may be pre-designed to facilitate interpretation from the desired perspective. In such situations, graphs may be preferable to tables. On the other hand, sometimes it is desirable to avoid favoring any particular perspective on the data over others, as is often the case when the primary goal is exploratory analysis or discussion. In such cases, graphical format might introduce an undesirable bias in interpretation of the data, and tabular format might, therefore, be preferable. Another viable approach to presenting data for exploratory purposes would be to create multiple graphs employing different assignments of visual dimensions to variables. While each individual graph might induce a biased perspective toward the data, any given perspective could be highlighted by at least one of the graphs.

The findings regarding flexibility also have implications regarding the respective design demands of graphs and tables. In the design of representations for bivariate data, even after a representational format is chosen, additional design choices are necessary, such as the decision of which variable to represent in the graph legend or table rows, and which to represent in the x-axis or table columns. The present findings suggest that such design decisions may be more consequential for graphs than for tables, in the sense that each possible decision will facilitate some tasks at the expense of others to a greater degree for graphs than for tables. A practical implication is that designing graphs may demand greater care than designing tables. However, the finding that graphs do support more efficient processing than tables overall suggests that the additional effort necessary for their design may be effort well-spent.

### Summary

Line graphs appear to be more efficient, but also less flexible, than tables for tasks involving detection of statistical effects in bivariate data. In particular, graphs facilitate detection of effects of the legend variable relative to those of the x-axis variable, while both effects are equally easy to detect with tables. The results suggest that graphs may be a preferable format for presenting data in order to emphasize a certain perspective, but greater care must be taken in the use of graphs when an unbiased presentation is desired.

### Conflict of interest statement

The authors declare that the research was conducted in the absence of any commercial or financial relationships that could be construed as a potential conflict of interest.

## Footnotes

^1^If the “v” is vertically symmetric, a secondary effect is not present, but if vertically asymmetric, a secondary effect is likely present.

^2^For simplicity, the issue of variance was not raised during the tutorials. In other words, participants were instructed to judge the presence or absence of effects based on the mean values alone. The standard that a difference was to be deemed large if greater than or equal to 5, and small otherwise, was used throughout both experiments.

^3^Differences in the salience of different effects is one possible explanation for influence from irrelevant effects, as described above. An alternate explanation is that the different effects were difficult to disentangle psychologically, resembling integral rather than separable dimensions (Goldstone and Steyvers, 2001; Jones and Goldstone, 2013). If this explanation is correct, both relevant and irrelevant effects should exert similar influence on a given task, i.e. *I_t,t_*≈ *I_x,t_* for *x* ≠ *t*. To test this possibility, influence from relevant effects (shown in the on-diagonal cells of Figure 4) was compared to influence from irrelevant effects (shown in the off-diagonal cells) separately for each task using Wilcoxon signed rank tests, using a Bonferroni correction for multiple comparisons. Influence from relevant effects was significantly greater for both the treatment and interaction tasks, *p*s < 0.001, although this was not true for the secondary task, *p* > 0.95. Additionally, if influence from irrelevant effects is caused by inseparability of the effects, such influence should be symmetric, i.e., *I_x,t_*≈ *I_t,x_* for *x* ≠ *t*. In other words, the values in the off-diagonal cells of Figure 4 should be equal to their reflections across the diagonal. To the contrary, influence from the treatment and interaction effects on the secondary task was greater than influences from the secondary effect on, respectively, the treatment and interaction tasks according to Wilcoxon signed rank tests, *p* s < 0.006, although influence from treatment effects on the interaction task did not differ from influence from interaction effects on the treatment task, *p* > 0.95. Thus, the patterns of influence are not consistent with psychological inseparability of different effects.

^4^For graphical stimuli, influence *Ix, t* for *x* = “interaction” in Experiment 1 was 25.7% for *t* = “treatment” and 26.7% for *t* = “secondary,” and in Experiment 2 was 25.3% for *t* = “treatment” and 27.3% for *t* = “secondary.” Influence of interaction effects on responses to the treatment and secondary tasks did not differ in either experiment, *p* s > 0.75.
