# Supplementary material for: Flexibility in data interpretation: effects of representational format
Source: Front Psychol. 2013 Dec 31;4:980. doi: 10.3389/fpsyg.2013.00980 (PMC3876463; doi:10.3389/fpsyg.2013.00980)
Supplement: Supplementary file 1 [file DataSheet1.DOCX]

# Supplementary Materials for

# Flexibility in data interpretation: Effects of representational format

David W. Braithwaite*^[[1]](#footnote-1)^, Robert L. Goldstone^2^

**Correspondence:**

David W. Braithwaite

1101 E. 10th Street

Bloomington, IN 47405

baixiwei@gmail.com

## Section A: Learning with Multiple Representations

The Introduction of the primary manuscript discusses the possibility of transfer effects, either positive or negative, between different tasks performed with the same representational format. The general concept of transfer also includes positive or negative effects of training between different representational formats with the same task, which presumably requires an understanding of the task structure that is not tied to the details of a particular format (for a taxonomy of types of transfer, see Barnett and Ceci, 2002). Positive transfer of both types is desirable from a pedagogical perspective. For example, a statistics instructor presumably hopes that students who have learned to detect main effects of drink flavor with graphs and tables will also be able to detect not only main effects of age group in the same graphs and tables, but also main effects of both variables in a novel representational format, such as text passages. However, such transfer of training is notoriously difficult to achieve, and numerous studies have found that even small differences between training and transfer contexts can cause failure to transfer (Catrambone & Holyoak, 1989; Detterman, 1993; Gick & Holyoak, 1980).

Educational theories suggest that instruction involving multiple external representations, e.g. both tables and graphs, is one way to promote transfer. Presumably, exposure to multiple external representations can lead learners to integrate information from these external representations, thus forming more robust and general internal concepts than could be derived from single external representations presented on their own (Ainsworth, 2006; Mayer, 2002; Schnotz, 2005). Such internal concepts should, in turn, be relatively easily applied to novel contexts and tasks, and thus promote positive transfer (Gick & Holyoak, 1983; Kotovsky & Gentner, 1996; Medin & Ross, 1989).

Consistent with this view, several studies have indeed found evidence for better learning outcomes following instructional interventions aimed at encouraging integration of information from multiple external representations (Berthold & Renkl, 2009; Booth & Siegler, 2008; Kellman, Massey, & Son, 2010; Rau, Aleven, & Rummel, 2009). One of the main tenets of the widely used Everyday Mathematics curriculum is that instruction with multiple representations is crucial for students’ development of flexible and deep knowledge of mathematics (Fuson, Carroll, & Drueck, 2000). However, if students are not encouraged to or unable to achieve such integration, exposure to multiple representations may be either not beneficial or even harmful (Berthold & Renkl, 2009; Kolloffel, Eysink, de Jong, & Wilhelm, 2008; Rau et al., 2009). These findings suggest that the effects of learning with multiple representations may depend on details of the instructional approach.

One approach that may have the potential to improve such effects is prompting learners to make explicit comparisons between different representations of the same information. Analogy research suggests that comparing analogous examples is a powerful tool to facilitate generalization and transfer. A classic result is that comparing isomorphic problems leads to a more abstract conception of their solutions, and consequently to more successful solution of analogous problems encountered subsequently (Gick & Holyoak, 1983). More recently, this finding has been replicated in a realistic educational setting (Gentner, Loewenstein, & Thompson, 2003). Management trainees who compared case studies illustrating a negotiation technique were more likely to apply the technique appropriately on a subsequent test than trainees who studied the cases separately, without comparing them. The standard theoretical account of these results is that the process of comparing examples increases the salience of their shared structure, while backgrounding superficial details that are idiosyncratic to one or the other example (Gentner, 2010). Importantly, this account is quite similar to that proposed above for the potential benefits of learning with multiple representations. This observation suggests that comparing different representations could deliver benefits analogous to those observed from comparing different examples, namely, better generalization and higher rates of transfer. However, there is little direct evidence for this possibility. One exception is a study of Rau et al. (2009), which found improved learning and transfer when students were prompted to explain connections between graphical and symbolic representations of fractions.

Although the studies just mentioned (Gentner et al., 2003; Gick & Holyoak, 1983; Rau et al., 2009) provide evidence that comparison between examples or alternate representations can improve transfer relative to no comparison, one might object that “no comparison” is a rather weak benchmark. Relative to no comparison, comparison may lead to additional effort, deeper processing, or longer study time, any or all of which might also result from other types of intervention and lead, in turn, to better learning outcomes even without the more general internal concepts hypothesized to result from comparison. From a theoretical point of view, this observation implies that the benefits of comparing examples or alternate representations may not be due entirely to the formation of more general internal concepts. From a practical point of view, it means that such comparison may not offer any incremental benefit in learning outcomes, relative to plausible alternatives involving the same materials and, crucially, requiring similar levels of learner engagement with those materials. From both points of view, then, a test against a more stringent benchmark would be desirable.

One possible benchmark is instruction in which learners are prompted to compare not isomorphic examples (Gentner et al., 2003; Gick & Holyoak, 1983) or alternate representations of the same information (Rau et al., 2009), but rather contrasting examples and counter-examples within a single representational format. Because examples and counter-examples, by definition, are structurally distinct, comparing them is likely to make their differences, rather than their similarities, more salient. Thus, the above account of the benefits of comparing isomorphic examples or representations is unlikely to apply to comparison of examples and counter-examples. Nevertheless, there is considerable evidence for the utility of studying contrasting cases (Gick & Paterson, 1992; Reed, 2006; Rittle-Johnson & Star, 2009; Schwartz & Bransford, 1998; Schwartz, Chase, Oppezzo, & Chin, 2011). Presumably, comparing contrasting cases leads to a better understanding of the relevant dimensions of variation in the domain, and how these dimensions affect the applicability of learned concepts. The present study compared learning outcomes following interventions based either on comparing isomorphic examples (or counter-examples) across representational format, or on comparing examples and counter-examples within one representational format. Because both forms of comparison are potentially beneficial, a no-comparison condition was also included as a benchmark.

### Methods

Participants in the experiments described in the primary manuscript were randomly assigned to one of three training conditions: comparing representations, contrasting examples, or control. These training conditions were implemented in the same way in Experiments 1-2, except for differences in the training and test materials, as described in the primary manuscript. In Experiment 1, N=42 participants were assigned to the comparing representations condition, N=41 to the contrasting examples condition, and N=44 to the control condition. In Experiment 2, N=14 participants were assigned to the comparing representations condition, N=15 to the contrasting examples condition, and N=14 to the control condition.

During each of the tutorials regarding treatment, interaction, and secondary effects described in the primary manuscript, participants were presented with the four training examples for the given effect in two pairs, with the two examples within a pair being presented side by side. After determining whether the given effect was present in either or both of these examples, participants were then asked to compare the two examples using an open response format. This procedure was then repeated for the second pair of training examples for the given effect.

Which training examples were shown together, and therefore compared by participants, varied according to training condition. In the comparing representations condition, the first two stimuli shown in each tutorial were the table and graph in which the effect in question was present, while the second two stimuli were the table and graph in which the effect was absent. Thus, participants in this condition saw tables and graphs of the same data displayed side by side and were asked to compare these. In the contrasting examples condition, the first two stimuli shown were the tables of the data in which the effect in question was present and of the data in which it was absent, while the second two stimuli were the corresponding graphs. Thus, participants in this condition saw examples and counter-examples of the given effect displayed side by side within each representational format, and were asked to compare these. Finally, in the control condition, the first two stimuli shown were the table in which the effect in question was present and the graph in which it was absent, while the second two stimuli were the table in which it was absent and the graph in which it was present. The control condition was intended to provide the same amount of variation across training stimuli, but not to afford opportunities for potentially useful comparisons, as in the other two conditions. Importantly, participants in the control condition were *not* prompted to compare stimuli within each pair.

As described in the primary manuscript, in the tutorial for secondary effects, participants were not provided with any instruction or feedback regarding how to determine whether secondary effects are present. However, after completing all four training stimuli, participants were asked to explain, in open-response format, how they thought one could determine whether an effect of the secondary factor was present. This question served as a measure of how well participants could adapt their understanding of statistical effects from the previous two tutorial sections. Participants’ responses to this question were coded for correctness on a scale of 0 to 2. Responses indicating that the average scores for each level of the secondary factor should be calculated and then compared were assigned a score of 2. Responses indicating that the levels of the secondary factor should be compared, but not specifying that what should be compared is the average for each level of the secondary factor over levels of the treatment factor, were assigned a score of 1. Responses not involving comparing levels of the secondary factor, or indicating incorrectly that what should be compared is differences, rather than averages, over levels of the treatment factor, were assigned a score of 0. All responses were coded by two independent coders, one of whom was the first author, and all disagreements were resolved through discussion.

### Results

In Experiment 1, the main effect of training condition on test accuracy was not significant, nor were any of its interactions with other factors, *p*s>.35.

Responses to the open-response question in which participants were asked to describe how they thought one should determine whether a secondary effect was present were scored as described in the Method section, with 60 responses receiving scores of 0, 22 receiving scores of 1, and 45 receiving scores of 2. Scores were highly correlated with accuracy on the secondary task, Pearson’s *r*=.386, *t*(125)=4.68, *p*<.001, suggesting that these scores were a meaningful measure of learning. The number of responses receiving each score in each training condition are shown in Table 1. A Chi-squared test of independence was employed to determine whether training condition affected the distribution of scores. The result was not significant, χ^2^(4)=7.09, *p*=.131.

Table 1.

*Frequencies of Scores on Secondary Effect Open-Response Question by Experiment and Training Condition.*

|  | Experiment 1 | | |  | Experiment 2 | | |
| --- | --- | --- | --- | --- | --- | --- | --- |
| Score | Comp. Rep. | Contr. Ex. | Control |  | Comp. Rep. | Contr. Ex. | Control |
| 2 | 14 | 18 | 13 |  | 1 | 4 | 2 |
| 1 | 9 | 2 | 11 |  | 1 | 6 | 0 |
| 0 | 19 | 21 | 20 |  | 12 | 5 | 12 |
| Total | 42 | 41 | 44 |  | 14 | 15 | 14 |

In Experiment 2, neither the main effect of training condition on test accuracy, nor any of its interactions with other factors, reached significance, *p*s>.50.

Responses to the open-response question regarding how to determine whether a secondary effect was present were scored as in Experiment 1, with 29 responses receiving scores of 0, 7 receiving scores of 1, and 7 receiving scores of 2. As in Experiment 1, scores were highly correlated with accuracy on the secondary task, Pearson’s *r*=.369, *t*(41)=2.55, *p*=.015. The number of responses receiving each score in each training condition are shown in Table 1. A Chi-squared test of independence applied to the frequency table of score and training condition found a significant result, χ^2^(4)=13.90, *p*=.008, in contrast to Experiment 1. Better scores, i.e. scores of 2 and 1, were more frequent in the contrasting examples condition (10 out of 15 responses) than in the comparing representations (2 out of 14 responses) or control (2 out of 14 responses) conditions.

### Discussion

Instruction involving comparison between alternate representations of data illustrating the presence or absence of statistical effects did not lead to the predicted benefits of superior transfer to a novel representational format (i.e. text) or to a novel task (i.e. the secondary task), either when compared to a plausible alternative instructional approach, i.e. contrasting examples, or to a control condition. This negative finding does not contradict the extensive literature demonstrating that comparison between examples that possess the same structure, but differ in semantic detail, can promote subsequent transfer (Gentner et al., 2003; Gick & Holyoak, 1983). However, it does call into question whether similar benefits accrue to comparison between different representations of a single example.

Several educational theories propose that learners can benefit from integrating information from multiple representations (Ainsworth, 2006; Mayer, 2002; Schnotz, 2005). The absence of an advantage for the comparing representations condition of the present study suggests either that comparing representations did not induce such integration, or that such integration did not yield the expected benefits. It is possible, of course, that learning from multiple representations offers other types of benefits not detected by the present study, which did not include a “single representation” training condition. It is also possible that integration could be more effectively encouraged by methods other than comparing alternate representations. A separate study not reported here tested the effectiveness of encouraging integration by requiring participants to match, rather than compare, equivalent graphs and tables. While this method has produced positive learning outcomes in other settings (Kellman et al., 2010), it produced no advantage over control in the present paradigm.

Experiment 2 did find some evidence of a benefit from another experimental condition, contrasting examples. Participants in this condition were relatively likely to derive a correct method for performing the secondary task after receiving training in the treatment and interaction tasks. Although this advantage was not reflected in participants’ performance on the test regarding the secondary task, it suggests that the contrasting examples condition may at least have laid a better foundation for understanding that task. Thus, the contrasting examples condition might be expected to lead to superior learning from subsequent explicit instruction regarding the secondary task (Schwartz & Martin, 2004). However, this prediction is not directly supported by our data.

At least two factors may have contributed to the advantage of contrasting examples. First, comparing multiple examples within each format may have promoted representational fluency (Rau, Aleven, & Rummel, 2010). Second, comparing contrasting examples may have deepened participants’ understanding of the principles of the domain by highlighting relevant dimensions of variation between examples (Gick & Paterson, 1992; Reed, 2006; Schwartz & Bransford, 1998; Schwartz et al., 2011). Of course, these two factors are not mutually exclusive, and may both have played some role.

### References

Ainsworth, S. (2006). DeFT: A conceptual framework for considering learning with multiple representations. *Learning and Instruction*, *16*(3), 183–198.

Barnett, S. M., & Ceci, S. J. (2002). When and where do we apply what we learn? A taxonomy for far transfer. *Psychological Bulletin*, *128*(4), 612–637.

Berthold, K., & Renkl, A. (2009). Instructional aids to support a conceptual understanding of multiple representations. *Journal of Educational Psychology*, *101*(1), 70–87. doi:10.1037/a0013247

Booth, J. L., & Siegler, R. S. (2008). Numerical magnitude representations influence arithmetic learning. *Child Development*, *79*(4), 1016–1031.

Catrambone, R., & Holyoak, K. J. (1989). Overcoming contextual limitations on problem-solving transfer. *Journal of Experimental Psychology: Learning, Memory, and Cognition*, *15*(6), 1147–1156. doi:10.1037//0278-7393.15.6.1147

Detterman, D. K. (1993). The case for the prosecution: transfer as an epiphenomenon. In D. K. Detterman & R. J. Sternberg (Eds.), *Transfer on trial: Intelligence, cognition, and instruction* (pp. 1–24). Norwood, NJ: Ablex.

Fuson, K., Carroll, W., & Drueck, J. (2000). Achievement results for second and third graders using the Standards-based curriculum Everyday Mathematics. *Journal for Research in Mathematics Education*, *31*(3), 277–295.

Gentner, D. (2010). Bootstrapping the mind: Analogical processes and symbol systems. *Cognitive Science*, *34*(5), 752–75. doi:10.1111/j.1551-6709.2010.01114.x

Gentner, D., Loewenstein, J., & Thompson, L. (2003). Learning and transfer: A general role for analogical encoding. *Journal of Educational Psychology*, *95*(2), 393–405. doi:10.1037/0022-0663.95.2.393

Gick, M. L., & Holyoak, K. J. (1980). Analogical problem solving. *Cognitive Psychology*, *12*, 306–355.

Gick, M. L., & Holyoak, K. J. (1983). Schema induction and analogical transfer. *Cognitive Psychology*, *15*, 1–38.

Gick, M. L., & Paterson, K. (1992). Do contrasting examples facilitate schema acquisition and analogical transfer? *Canadian Journal of Psychology*, *46*(4), 539–550.

Kellman, P. J., Massey, C. M., & Son, J. Y. (2010). Perceptual learning modules in mathematics: Enhancing students’ pattern recognition, structure extraction, and fluency. *Topics in Cognitive Science*, *2*(2), 285–305. doi:10.1111/j.1756-8765.2009.01053.x

Kolloffel, B., Eysink, T. H. S., de Jong, T., & Wilhelm, P. (2008). The effects of representational format on learning combinatorics from an interactive computer simulation. *Instructional Science*, *37*(6), 503–517. doi:10.1007/s11251-008-9056-7

Kotovsky, L., & Gentner, D. (1996). Comparison and categorization in the development of relational similarity. *Child Development*, *67*(6), 2797–2822.

Mayer, R. E. (2002). Multimedia learning. *The Psychology of Learning and Motivation*, *41*, 85–139. doi:10.1017/CBO9781139164603

Medin, D. L., & Ross, B. H. (1989). The specific character of abstract thought: Categorization, problem-solving, and induction. In R. J. Sternberg (Ed.), *Advances in the psychology of human intelligence, Vol. 5* (pp. 189–223). Hillsdale, N.J.: Lawrence Erlbaum Associates, Inc.

Rau, M. A., Aleven, V., & Rummel, N. (2009). Intelligent tutoring systems with multiple representations and self-explanation prompts support learning of fractions. In V. Dimitrova, R. Mizoguchi, & B. du Boulay (Eds.), *Proceedings of the 14th International Conference on Artificial Intelligence in Education* (pp. 441–448). Amsterdam, the Netherlands: IOS Press.

Rau, M. A., Aleven, V., & Rummel, N. (2010). Blocked versus interleaved practice with multiple representations in an intelligent tutoring system for fractions. In V. Aleven, J. Kay, & J. Mostow (Eds.), *Intelligent tutoring systems* (pp. 413–422). Berlin / Heidelberg: Springer.

Reed, S. K. (2006). Does unit analysis help students construct equations? *Cognition and Instruction*, *24*(3), 341–366. doi:10.1207/s1532690xci2403_2

Rittle-Johnson, B., & Star, J. R. (2009). Compared with what? The effects of different comparisons on conceptual knowledge and procedural flexibility for equation solving. *Journal of Educational Psychology*, *101*(3), 529–544. doi:10.1037/a0014224

Schnotz, W. (2005). An integrated model of text and picture comprehension. In *The Cambridge handbook of multimedia learning*. Cambridge, UK: Cambridge University Press.

Schwartz, D. L., & Bransford, J. (1998). A time for telling. *Cognition and Instruction*, *16*(4), 475–522.

Schwartz, D. L., Chase, C. C., Oppezzo, M. A., & Chin, D. B. (2011). Practicing versus inventing with contrasting cases: The effects of telling first on learning and transfer. *Journal of Educational Psychology*, *103*(4), 759–775. doi:10.1037/a0025140

Schwartz, D. L., & Martin, T. (2004). Inventing to prepare for future learning: The hidden efficiency of encouraging original student production in statistics instruction. *Cognition and Instruction*, *22*(2), 129–184.

## Section B: Complete Test Stimuli (Experiment 1)

| Effects Present | Graph | Table | Text |
| --- | --- | --- | --- |
| None | 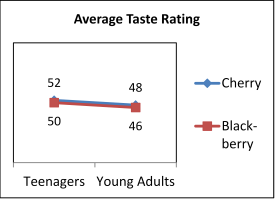 | 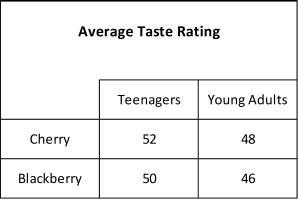 | 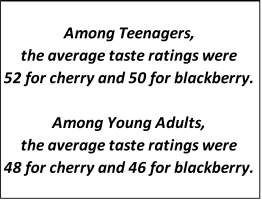 |
| None | 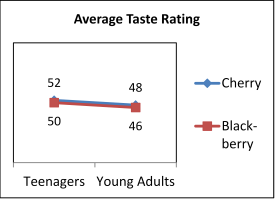 | 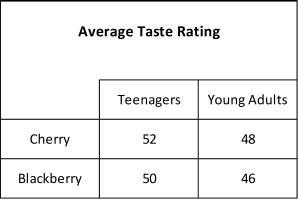 | 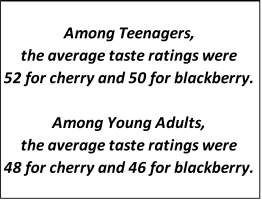 |
| Treatment | 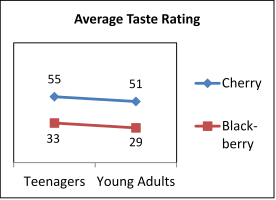 | 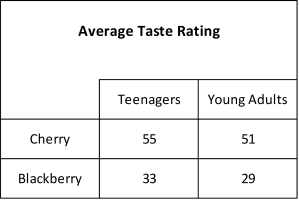 | 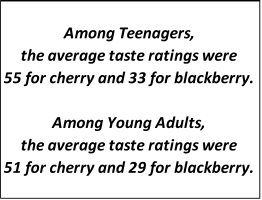 |
| Treatment | 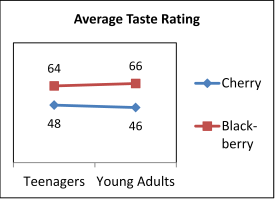 | 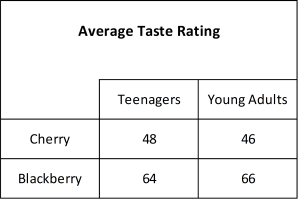 | 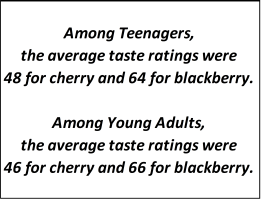 |
| Interaction | 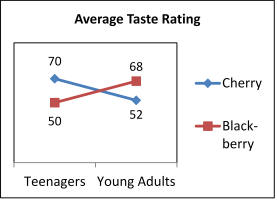 | 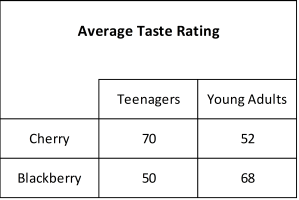 | 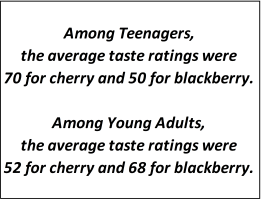 |
| Interaction | 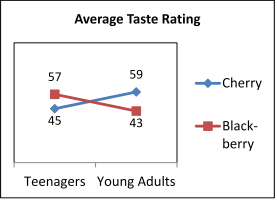 | 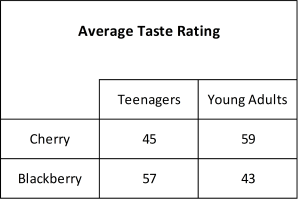 | 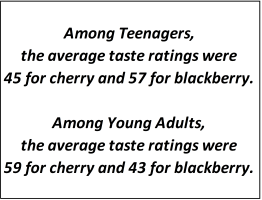 |
| Secondary | 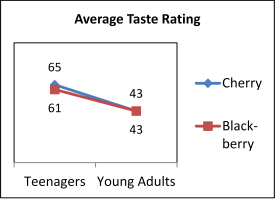 | 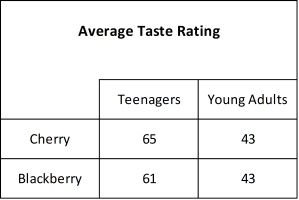 | 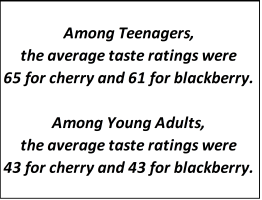 |
| Secondary | 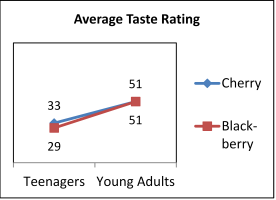 | 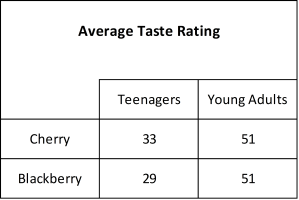 | 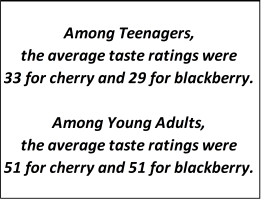 |
| Treatment,  Interaction | 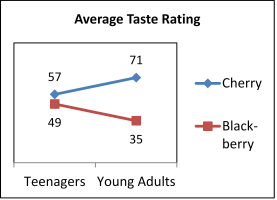 | 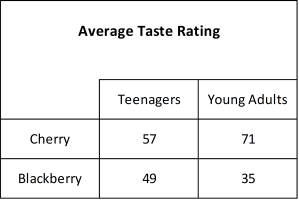 | 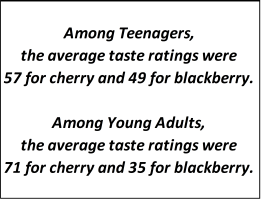 |
| Treatment,  Interaction | 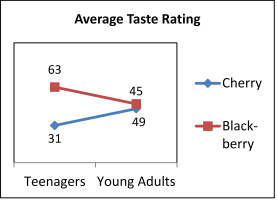 | 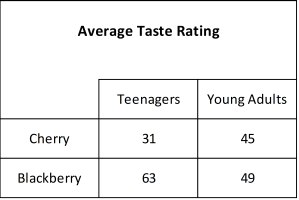 | 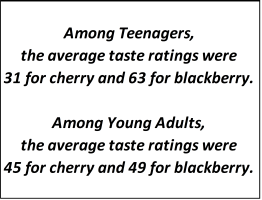 |
| Treatment,  Secondary | 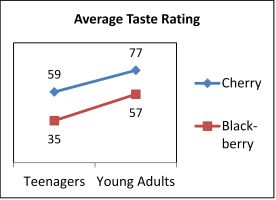 | 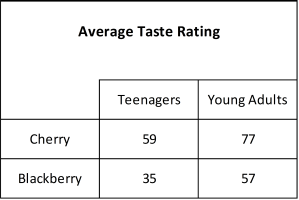 | 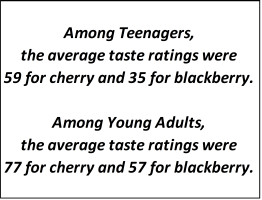 |
| Treatment,  Secondary | 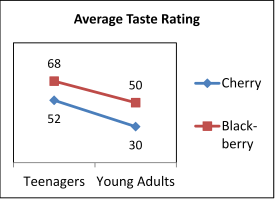 | 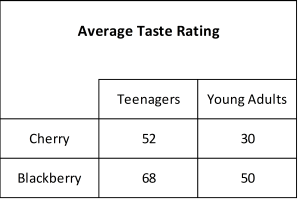 | 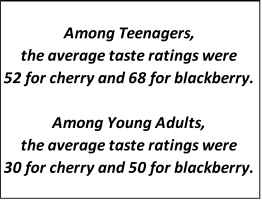 |
| Interaction,  Secondary | 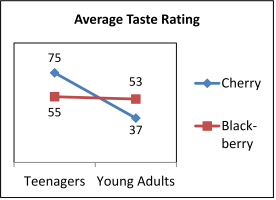 | 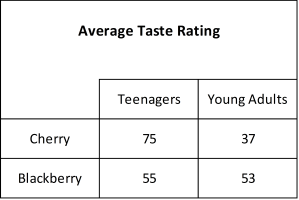 | 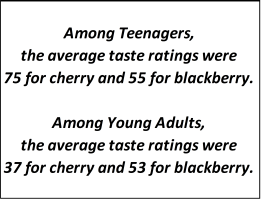 |
| Interaction,  Secondary | 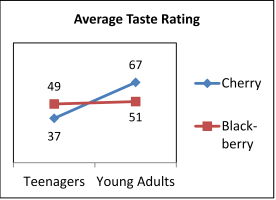 | 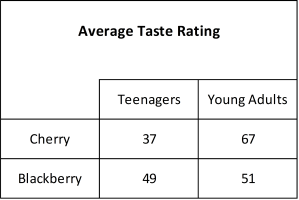 | 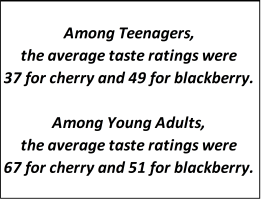 |
| Treatment,  Interaction,  Secondary | 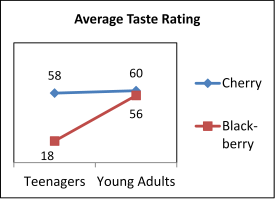 | 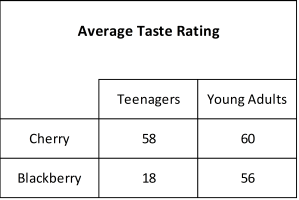 | 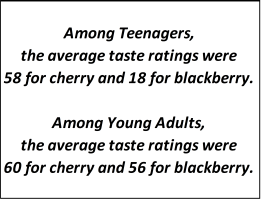 |
| Treatment,  Interaction,  Secondary | 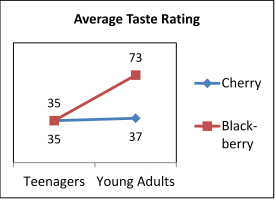 | 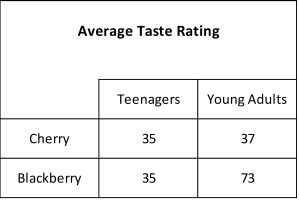 | 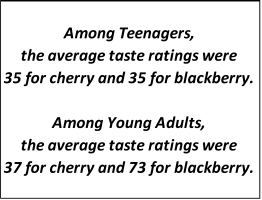 |

## Section C: Complete Test Stimuli (Experiment 2)

| Effects Present | Graph | Table | Text |
| --- | --- | --- | --- |
| None | 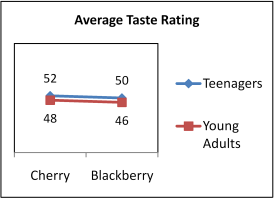 | 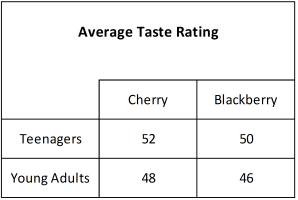 | 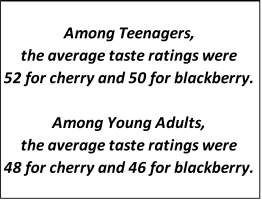 |
| None | 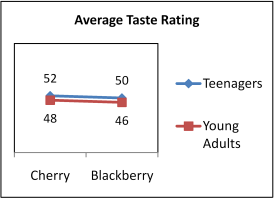 | 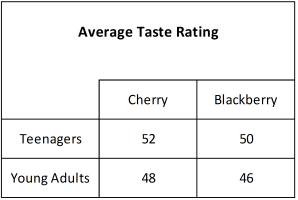 | 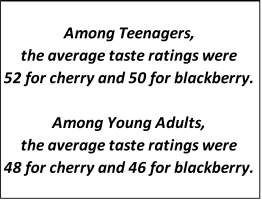 |
| Treatment | 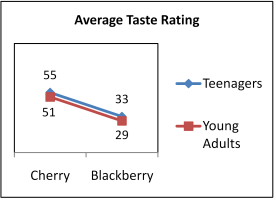 | 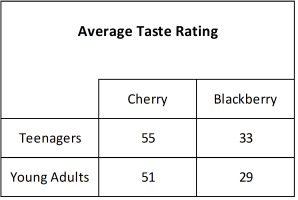 | 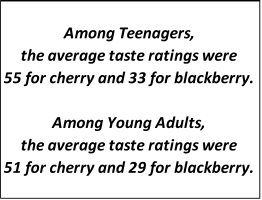 |
| Treatment | 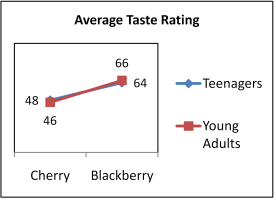 | 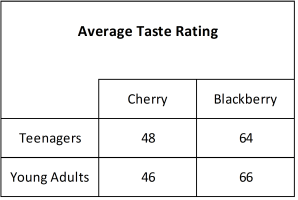 | 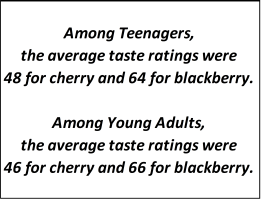 |
| Interaction | 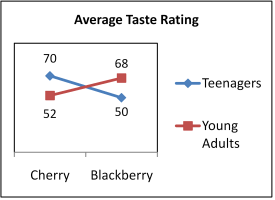 | 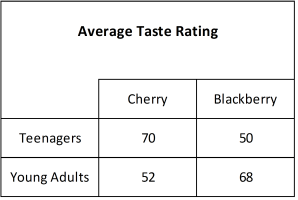 | 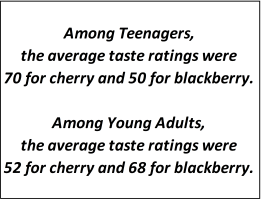 |
| Interaction | 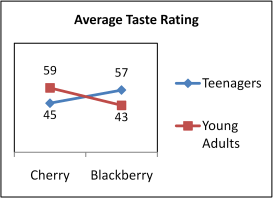 | 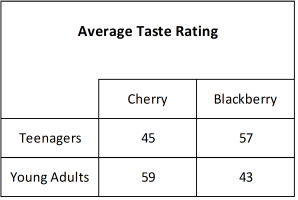 | 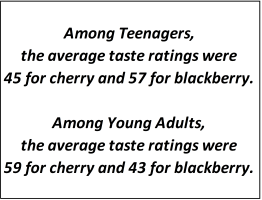 |
| Secondary | 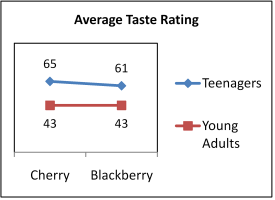 | 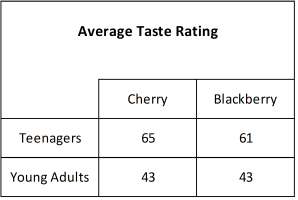 | 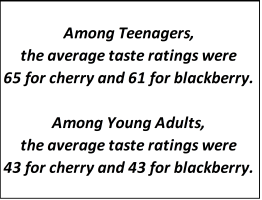 |
| Secondary | 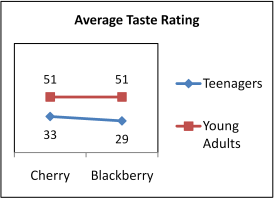 | 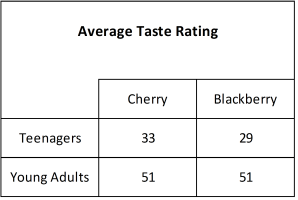 | 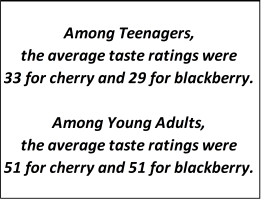 |
| Treatment,  Interaction | 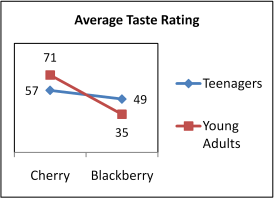 | 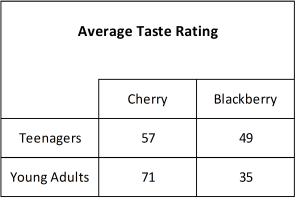 | 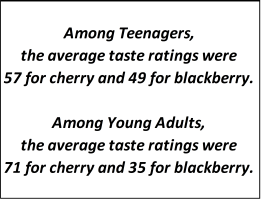 |
| Treatment,  Interaction | 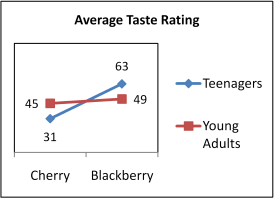 | 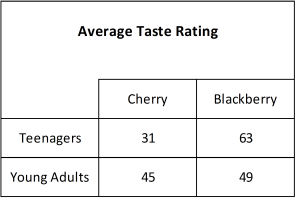 | 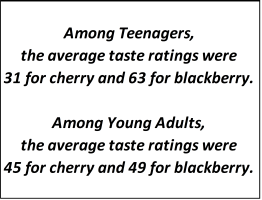 |
| Treatment,  Secondary | 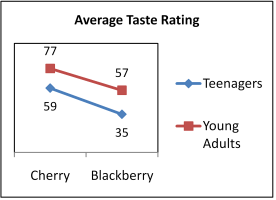 | 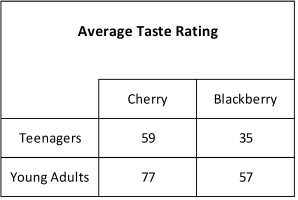 | 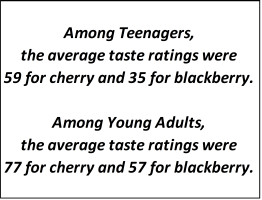 |
| Treatment,  Secondary | 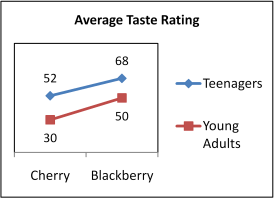 | 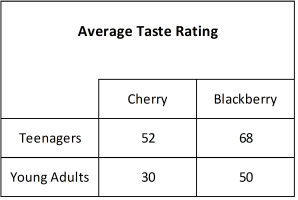 | 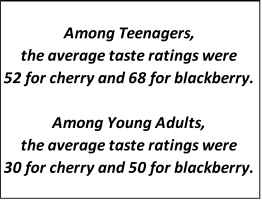 |
| Interaction,  Secondary | 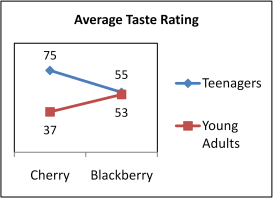 | 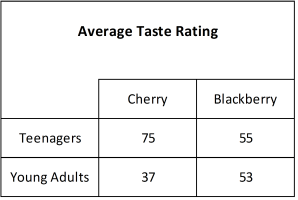 | 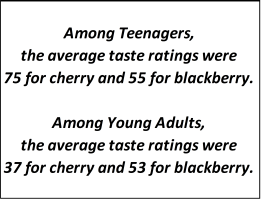 |
| Interaction,  Secondary | 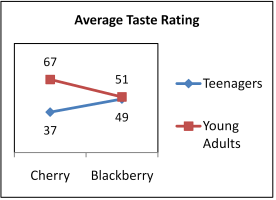 | 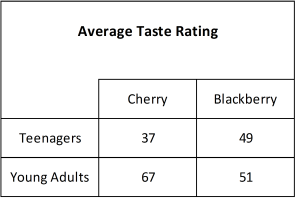 | 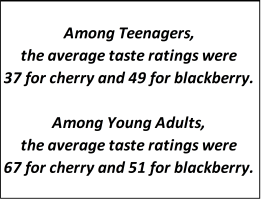 |
| Treatment,  Interaction,  Secondary | 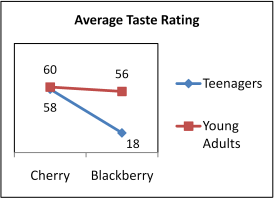 | 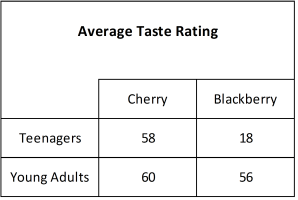 | 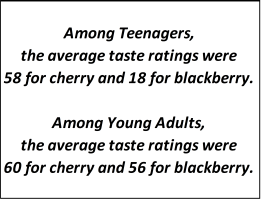 |
| Treatment,  Interaction,  Secondary | 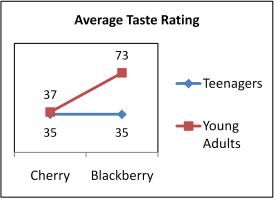 | 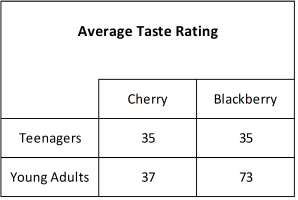 | 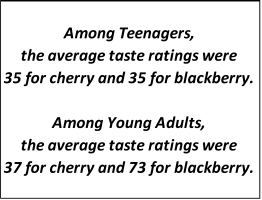 |

1. ^, 2^ Percepts-Concepts Laboratory, Department of Psychological and Brain Sciences, Indiana University, Bloomington, IN, USA. [↑](#footnote-ref-1)
